# A Critical Review of Knowledge Gaps in PGPR‐Based Enhancement of Medicinal Plant Production

**DOI:** 10.1155/sci5/3500228

**Published:** 2026-10-28

**Authors:** Oluwafunbi Christianah Adeleye, Noluyolo Nogemane

**Affiliations:** ^1^ Department of Agriculture and Animal Health College of Agriculture and Environmental Sciences University of South Africa Florida Campus Johannesburg South Africa unisa.ac.za

**Keywords:** *Bacillus*, bioactivity, medicinal plants, metabolomics, PGPR, *Pseudomonas*, secondary metabolites

## Abstract

The therapeutic value of medicinal plants is attributed to the accumulation and constituents of secondary metabolites, such as phenolics, flavonoids, and terpenoids. Plant growth‐promoting rhizobacteria (PGPR) provide an efficient approach to enhancing plant growth, development, health, and production. PGPR genera including *Bacillus, Streptomyces*, and *Pseudomonas* stimulate plant growth and development through a variety of mechanisms, including increased secondary metabolites, phytohormone production, and phytopathogen biocontrol, and thus promote sustainable agriculture. Africa is abundant in medicinal plants; however, the underrepresentation of PGPR research in Africa and the impact of specific environmental stress factors, such as drought, poor soil fertility, and climate variability, make PGPR‐based sustainable cultivation strategies highly relevant. Medicinal plants are therapeutic in nature and, similar to crop plants, are susceptible to environmental stressors. Research optimizing production in both quality and quantity of these plants is essential, and hence, PGPR‐mediated mechanisms that may enhance plant growth, stress tolerance, nutrient uptake, and sustainable cultivation practices are of interest. This review seeks to identify the gaps and limitations in recently published articles that focus on the application and influence of PGPR on the production of medicinal plants to inform future research on these rhizobacteria as well as optimize the use of PGPR. To collect relevant data, a variety of databases were used for literature search, such as Scopus, PubMed, ScienceDirect, and Web of Science. Several studies have demonstrated the beneficial effects of PGPR on the biosynthesis and accumulation of secondary metabolites in medicinal plants. The review of these studies revealed major limitations, including limited research conducted within Africa as well as a lack of validation of the bioactivity of the identified secondary metabolites. To address the limitations mentioned, it is important to conduct more continent‐based research on medicinal plants with a focus on the application of PGPR in Africa.

## 1. Introduction

Plants that contain compounds used for therapeutic purposes and serve as building blocks in the production of pharmaceuticals are known as medicinal plants. These plants also serve as a traditional method of healing, as they contain a variety of bioactive substances, including vitamins, aldehydes, anthocyanins, flavonoids, tannins, terpenoids, alkaloids, and phenolic compounds [1], which increase pharmacological properties such as antiviral, anticancer, antifungal, anti‐inflammatory, and antimalarial [2]. The therapeutic efficacy, simplicity of access, affordability, and minimal or nonexistent side effects compared to the negative effects of synthetic medications continue to make the use of medicinal plants more popular. More than 80% of the world’s population relies on some form of traditional medicine, which encompasses herbal therapies and other therapeutic practices [3]. Medicinal plants are used to manage a wide range of diseases and health conditions, including, but not limited to, infertility, diabetes, hepatitis, ulcers, piles, and dermatological disorders [4, 5].

The growth and development of plants are significantly influenced by their surroundings, and fundamental environmental elements such as light, temperature, water, humidity, and nutrition all have an impact on plant growth [1]. Despite this, unfavorable environmental circumstances lead to stress in plants, weakening them and increasing their vulnerability to pests and diseases. Abiotic stress causes morphological, anatomical, biochemical, and molecular responses in plants. Both primary and secondary metabolites are formed by biochemical mechanisms, and to survive adverse environmental conditions, plants synthesize a number of secondary metabolites as part of their biochemical response [6]. Environmental challenges expose medicinal plants to a variety of physiological changes and alterations in their chemical composition. Moreover, abiotic stressors activate plants’ detoxification mechanisms, which include increased synthesis of antioxidant enzymes such as superoxide dismutase (SOD), catalase (CAT), ascorbate peroxidase (APX), and glutathione peroxidase (POX) [7]. Flavonoids and lignin precursors are examples of phenolic compounds that accumulate in plants in response to abiotic stresses and are thus regarded as the fundamental processes for scavenging reactive oxygen species (ROS) [8]. The altered physiological and biochemical properties of medicinal plants by a variety of antagonistic abiotic factors then lead to the loss of their therapeutic value [1]. Challenges such as these will persist in the production of medicinal plants due to climate change and future predictions of extreme temperature events [9].

Notably, although plants have several coping mechanisms to deal with stress, high‐stress conditions have a negative impact on plants and can eventually lead to plant death and eventually become extinct. Thus, some techniques are used to boost plant stress tolerance to guarantee plant survival during adverse conditions [10]. The use of microorganisms as a biological strategy is one of the current initiatives used to increase stress tolerance in plants. For instance, in harsh environments, plants form mutually beneficial symbiotic interactions with these microorganisms [10], and the primary role of these microorganisms is to promote plant stress tolerance through hormone production (cytokinins, gibberellins, indole‐3‐acetic acid [IAA]), siderophore formation, phosphate solubilization, nutrient uptake, and pathogen antagonism [11].

Plant growth‐promoting rhizobacteria (PGPR) have been reported in numerous studies to mitigate the effects of adverse environmental conditions that significantly restrict plant growth and productivity, such as salinity, drought, and heat stress [12]. PGPRs use many ways to mitigate these antagonistic abiotic factors. For instance, PGPR‐induced production of exopolysaccharides (EPSs) can facilitate drought tolerance by preserving the soil moisture around roots [13]. Also, several PGPRs increase plant antioxidant systems, which reduce oxidative damage when water is scarce [12]. In saline environments, PGPRs help maintain ion homeostasis by controlling Na+/K+ ratios and encouraging the build‐up of osmolytes such as proline and glycine betaine in plant tissues [14]. Furthermore, PGPRs produce 1‐aminocyclopropane‐1‐carboxylate deaminase (ACC deaminase) in saline soils, which is advantageous since it lessens the inhibition of root development caused by excess ethylene (ET) production [14].

Africa is universally believed to be a climate change hotspot due to anthropogenic activities with little capacity for adaptation planning [15]. It is estimated that in 2039, heat waves would be more intense, particularly in the northern and eastern provinces of South Africa [16]. To meet current and future demands for medicinal plant‐based products, it is crucial to enhance stress resilience in African medicinal plants under adverse abiotic conditions. PGPRs have emerged as promising biological agents that improve plant growth and stress tolerance, while multiomics approaches offer valuable insights into the molecular and metabolic mechanisms governing plant–microbe interactions, thereby supporting the development of effective and sustainable PGPR‐based strategies [17]. By combining multiomics technologies such as transcriptomics, proteomics, metabolomics, genomics, metagenomics, and epigenomics, researchers have been able to better understand the complexity of plant–microbe interactions [18]. These technologies enable researchers to map protein–protein interactions; examine metabolic pathways that support symbiosis, defense, and tolerance for abiotic stresses; characterize gene expression variations in host plants; and decipher the genetic blueprints of PGPRs. Genomic investigations have made it possible to identify key characteristics of microorganisms that promote plant growth, such as nitrogen fixation, siderophore production, and stress‐responsive gene clusters [19]. Simultaneously, transcriptomic profiling has provided insights into hormonal regulation and plant immune priming under microbial control. In addition, proteomic analyses have revealed protein networks involved in microbial colonization, stress adaptation, and metabolite exchange, while specific markers such as ACC deaminase and antioxidant enzymes have been associated with plant growth promotion [20]. Lastly, metabolomics has revealed additional biochemical pathways influenced by plant growth‐promoting microorganisms (PGPMs), including enhanced accumulation of flavonoids, osmoprotectants, and signaling molecules in medicinal plants [18]. These omics‐based insights have facilitated the development of microbial consortia inoculants, which are often reported to be more effective than single‐strain inoculants in improving plant biomass, phytochemical accumulation, and stress tolerance [21].

Globally, more than 5000 research articles on medicinal plants are published annually [22]. According to Mahana [23], 3000 wild species have already been discovered for their therapeutic qualities. These medicinal plants are a primary source of topical cosmeceuticals and nutraceuticals containing bioactive ingredients that offer dermatological, nutritional, and overall health benefits [24]. Recently, there has been an increase in studies that seek to understand the role of rhizospheric and endophytic microorganisms in the production of medicinal plants and their secondary metabolites. Although PGPR‐mediated tolerance to abiotic and biotic stress has been widely studied in agricultural crops, the majority of this research originates from countries such as China, India, the United States, and Germany [25]. In contrast, African research remains limited and is primarily restricted to preliminary studies conducted in countries including Nigeria, South Africa, and Egypt. As a result, the application of PGPRs in medicinal plant production is still poorly explored in Africa despite the continent’s rich medicinal plant biodiversity and increasing climate‐related agricultural challenges.

In Africa, traditional healthcare systems and pharmaceutical applications utilize thousands of indigenous plant species [26]. Most of these plant species are endemic and adapted to extreme environmental conditions, making them valuable candidates for PGPR‐based studies to improve plant growth, stress tolerance, and secondary metabolite production [27]. Furthermore, African soils are associated with unique problems that can affect the productivity of medicinal plants and microbial interactions [28]. For instance, large areas of nutrient‐deficient soils with low organic matter, poor water‐holding capacity, salinity in arid regions, soil acidity, and land degradation from unsustainable agricultural practices exist in sub‐Saharan Africa [28]. These soil constraints can limit the growth and secondary metabolite accumulation of medicinal plants [29]. Moreover, Africa is highly vulnerable to climate change‐related stressors, such as recurrent droughts, erratic rainfall, prolonged heatwaves, desertification, and water scarcity [28]. These climatic stressors also adversely affect the establishment and biomass accumulation of medicinal plants [14]. Therefore, PGPRs with the potential to fix nitrogen, solubilize phosphates, produce siderophores, synthesize phytohormones, and exhibit ACC deaminase activity could be crucial in improving nutrient uptake, root growth, and abiotic stress tolerance under African climatic conditions [29]. Thus, the application of PGPRs may offer an environmentally sustainable approach to enhance the resilience and productivity of medicinal plants under climate‐stressed environments [12].

Several African medicinal plants, including species that have already been studied using PGPR, as well as underexplored taxa with high biotechnological potential, should be identified as priorities for future PGPR research. Some African medicinal plant species currently studied and considered promising for further PGPR investigations include *Artemisia afra*, which is highly valued for its pharmacological properties [30], and whose drought tolerance and nutrient enhancement could be improved through PGPR application. Also, *Hypoxis hemerocallidea* is a significant medicinal species in Southern Africa facing increasing challenges in cultivation and conservation [31], which could potentially be overcome by microbial‐assisted growth promotion. *Aloe ferox*, on the other hand, is a plant adapted to arid conditions, which makes it an ideal species for studying PGPR interaction in water‐deficit conditions [32]. Other promising species are *Lessertia frutescens*, which is prized for its ethnomedicinal significance [33], and *Warburgia salutaris*, an endangered medicinal species that may benefit from PGPR‐assisted conservation and sustainable cultivation approaches [34].

Thus, future studies should focus on African medicinal plant–PGPR interactions to address region‐specific environmental challenges, improve sustainable medicinal plant production, and strengthen climate‐resilient agricultural systems in Africa. Table 1 highlights some African medicinal plants with high PGPR research potential.

**TABLE 1:** Selected African medicinal plants with high PGPR research potential, including previously investigated species.

S/n	Medicinal plant	PGPR research potential	Importance	Reference
1.	*Warburgia salutaris*	PGPR may support conservation cultivation.	Endangered species, high medicinal value	[35]
2.	*Aloe ferox*	Useful for drought‐related PGPR studies.	Useful for the production of pharmaceuticals and cosmeceuticals for commercial purposes.	[32]
3.	*Artemisia afra*	Sensitive to drought and nutrient stress.	Used for traditional treatment of respiratory infections.	[30]
4.	*Greyia radlkoferi*	Little or no published work directly investigating PGPR inoculation in *Greyia radlkoferi.* Relatively drought‐tolerant, making it suitable for stress tolerance research.	High pharmaceutical and cosmeceutical properties. Potential for sustainable commercialization.	[36]
5.	*Lessertia frutescens*	Potential interaction with stress‐tolerant rhizobacteria.	Treatment of immune‐related conditions.	[33]
7.	*Portulacaria afra*	Adaptation to arid environments; tolerance to nutrient‐poor soils, and important for ecological restoration initiatives	High medicinal value	[37]
8.	*Hypoxis hemerocallidea*	Conservation and cultivation challenges.	High medicinal value	[31]
9.	Other Legumes, e.g., *Vachellia gummifera*	Adaptation to arid African environments. Potential for soil restoration and sustainable agriculture. Its leguminous characteristics.	Medicinal value	[38]

Given the large dependency on medicinal plant products and the predictions of climate change that can affect plant biodiversity and crop production in Africa, significant further research is needed on the role of the application of PGPRs in medicinal plants in the African context (Table 1). Therefore, the primary objectives of the present narrative‐critical review are to (1) analyze and synthesize current knowledge in PGPR‐based enhancement of medicinal plant production, (2) summarize the impacts of their application on secondary metabolite accumulation with associated bioactivities in medicinal plants, and (3) identify possible gaps and directions for future research endeavors in the formulation of more versatile or combined PGPR strains or a consortium of bacteria or strains to tackle concurrent abiotic factors in the production of medicinal plants.

## 2. Methodology

A structured literature search was conducted to inform the narrative‐critical synthesis of studies investigating the application of PGPR in medicinal plants. Relevant information was extracted from the published literature on the application of PGPR in medicinal plants, their effects on phytochemical composition and secondary metabolite accumulation, and associated biological activities, including antioxidant, antimicrobial, anti‐inflammatory, and other reported bioactivities. Electronic searches were conducted in Scopus, Web of Science, ScienceDirect, and PubMed using combinations of key concepts addressed in this review, including “plant growth‐promoting rhizobacteria” OR “PGPR,” “medicinal plants,” “secondary metabolites,” “phytochemicals,” “bioactivity,” and terms related to plant stress. The literature search focused on publications from 2020 to 2025. During revision, a limited number of studies published in 2026 were added to update specific aspects of the discussion. These studies were used as supporting literature and were not considered part of the original literature set defined by the 2020–2025 search period. The search period was selected to focus the review on recent research and emerging gaps in the application of PGPR in medicinal plants.

The narrative‐critical synthesis focused on studies that investigated PGPR, or closely related plant‐associated growth‐promoting bacteria, in medicinal plants and reported measurable effects on plant growth, stress responses, phytochemical composition, secondary metabolite accumulation, or biological activity. Retrieved records were initially screened by title and abstract, followed by examination of potentially relevant articles in full text. Literature was excluded when it fell outside the medicinal plant–PGPR focus or lacked sufficient methodological or outcome information to address the review objectives. Studies providing sufficient information to assess the experimental approach and reported outcomes were considered in the comparative synthesis. Covidence (Veritas Health Innovation, Melbourne, Australia) was used to organize the retrieved literature and facilitate the screening process.

The selected literature was critically appraised based on experimental design and the extent to which changes in secondary metabolites were investigated and linked to biological activity. Studies conducted under field conditions were considered to provide greater practical relevance because they incorporated environmental variability and cultivation conditions that may occur outside controlled environments; however, greenhouse and laboratory studies were also considered valuable for identifying controlled physiological and biochemical responses. Studies that quantified secondary metabolite accumulation and evaluated associated biological activities, such as antioxidant, antimicrobial, or anti‐inflammatory activity, were considered to provide stronger evidence for linking PGPR‐induced phytochemical changes with medicinally relevant outcomes. In contrast, studies reporting changes in metabolite abundance without corresponding biological activity assays were considered to provide more limited evidence regarding the functional significance of these changes.

## 3. Plant–Microbe Interaction

Plant–microbe interactions are complex, dynamic, and ongoing processes that date back to the origins of plant life. Plants and microorganisms have coexisted for millions of years, resulting in an assemblage of host and nonhost species that create a distinct ecological unit known as the “holobiont” [39]. Beneficial and pathogenic microorganisms, primarily bacteria and fungi, frequently invade plants in agricultural and natural environments [39]. The beneficial plant–microbe interactions are mediated through several direct and indirect mechanisms, including stimulation of plant growth through phytohormone production, antagonism against pathogenic microorganisms, stress mitigation, and nutrient transfer. Nutrient transfer is facilitated by mycorrhizal fungi and rhizobia, which are associated with plant roots and supply mineral nutrients and fixed nitrogen, respectively [40]. However, the negative interactions are toxic to plants because the invasive microorganisms may be saprophytic and cause necrotrophy in the colonizing plants [39]. As a result, understanding plant–microbe interactions is crucial for distinguishing the beneficial and negative effects of microbes on plants.

Plants interact with bacteria at three separate layers: the endosphere, phyllosphere, and rhizosphere [41]. The rhizosphere is an area where roots and soil come in contact. This region is a cloud of microorganisms that envelop plant roots and are critical to the plant’s survival and development [41]. Then, bacteria on or within plant tissue generate a variety of signals, including those based on proteins and carbohydrates, hormones and hormone mimics, and volatile organic compounds (VOCs). For instance, microbes have carbohydrate‐ and protein‐based signals known as microbe‐ or pathogen‐associated molecular patterns (MAMPs or PAMPs), which are necessary for microbial survival [42]. Also, plants develop distinct plasma membrane‐localized pattern recognition receptors (PRRs) that bind MAMPs and PAMPs, which regulate plant immune responses due to their conservation and the fact that they are not generated in plant cells. Plants respond to PAMPs by triggering a defense response known as PAMP‐triggered immunity (PTI) or basal resistance, which is the first line of defense that prevents pathogen invasion in most plant species [42]. During the invasion, microorganisms release effector molecules, which are critical components of pathogenesis [43]. Plants respond by developing resistance (R) genes that encode R proteins, allowing them to recognize, directly or indirectly, some of these effectors (avirulence proteins). Therefore, the recognition of a pathogen’s avirulence protein causes a set of immune responses known as effector‐triggered immunity (ETI) [43].

Recently, with the advent of high‐throughput genome sequencing of plants and microbes, innovative omics technologies have made it possible to better understand the mechanisms behind microbe detection, signal transduction, symbiosis formation, and plant defense responses [44].

## 4. PGPRs and Their Mechanisms of Action

Abiotic and biotic stress in medicinal plants is caused by rapid and gradual environmental changes, which eventually affect output [45]. Plants have developed diverse methods to cope with abiotic stressors such as salinity, drought, heavy metals, and temperature, which produce ROS, such as superoxide radical, hydroxyl radical, and hydrogen peroxide [46]. Using PGPR to mediate plant resilience to environmental challenges is one of these diverse methods [47].

PGPMs promote plant development, productivity, and health [48]. PGPRs, a subset of these microorganisms, are bacteria that inhabit the rhizosphere and enhance plant growth through direct and indirect mechanisms [17]. PGPRs encompass a diverse array of bacterial genera, including *Arthrobacter, Azotobacter, Azospirillum, Pseudomonas, Bacillus, Acetobacter, Micrococcus, Burkholderia, Paenibacillus, Agrobacterium, Caulobacter, Chromobacterium, Erwinia, Flavobacterium, Serratia, and Rhizobium.* Among these, *Azospirillum, Pseudomonas, and Bacillus*, along with various members of the phylum Actinobacteria, are among the most commonly used commercial PGPRs [49]. Most importantly, effective rhizosphere colonization is essential for the successful application of PGPRs in phytostimulation, biofertilization, phytoremediation, and biocontrol.

The mode of action of PGPRs occurs by means of direct and indirect mechanisms (Figure 1). In the indirect mechanism, PGPRs regulate plant pathogens, while in the direct mechanism, they speed up the uptake of nutrients from the environment or change plant growth by modifying hormone levels [50]. The direct mechanism includes biological nitrogen fixation, phytohormone synthesis (such as auxins, ET, cytokinins, and gibberellins), increased phosphorus and iron solubilization and availability, and the production of siderophores and ACC deaminase activity [46]. The indirect mechanism, on the other hand, includes antibiosis, Fe chelation in the rhizosphere, and the reduction of biotic and abiotic stresses, such as the promotion of induced systemic resistance (ISR), synthesis of fungistatic compounds (lipopeptides, polyketides), and hydrolytic enzymes that break down the cell walls of pathogens and plant pests [12]. However, depending on the plant species and bacterial strains used, these mechanisms may have different impacts [11].

**FIGURE 1:**
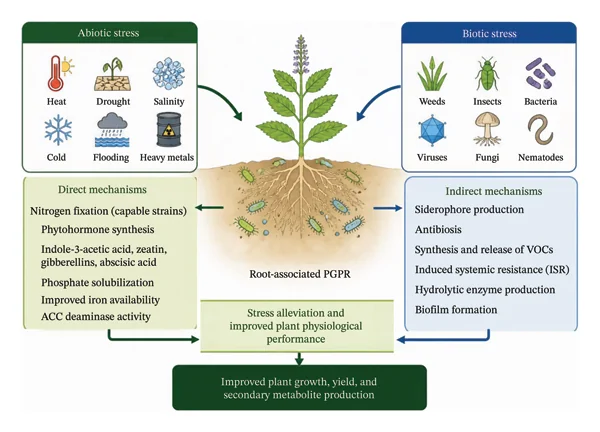
The role and mechanism of action of PGPR.

### 4.1. The Role of PGPRs in Medicinal Plants

#### 4.1.1. Disease Resistance

Plants can experience biotic stress from a variety of causes, such as weeds, nematodes, bacteria, viruses, and arachnids. Numerous biotic stressors have the potential to damage plants by weakening their defenses against various diseases and even causing plant mortality [12]. Plant growth and disease resistance can be improved with the application of PGPRs. For instance, siderophores that chelate iron, solubilize minerals, produce phytohormones, and synthesize compounds or enzymes that support plant growth are produced by these bacteria when they colonize plant roots [51]. Also, PGPRs shield plants from phytopathogens by vying for space and creating chelators that uniquely bind with Fe^3+^ ions, thereby limiting iron availability to phytopathogens [52]. PGPRs strengthen the immunity of medicinal plants via production of metabolites with immunostimulatory, insecticidal, antifungal, and antibiotic qualities [53]. The production of these many low‐molecular‐weight metabolites is primarily responsible for PGPRs’ capacity to generate plant immunity [53].

Several PGPR species, including *Bacillus subtilis*, *Pseudomonas fluorescens*, *Enterobacter asburiae*, and members of the Actinobacteria, contribute significantly to disease resistance in plants through multiple direct and indirect mechanisms [54]. PGPRs enhance plant defense responses by producing VOCs, EPSs, hydrolytic enzymes, antibiotics, and other antimicrobial metabolites that suppress phytopathogenic microorganisms [49]. In addition, PGPRs promote ISR, a defense mechanism triggered by nonpathogenic rhizobacteria that enhances plant protection against a broad spectrum of pathogens [55]. Unlike systemic acquired resistance (SAR), which is largely dependent on salicylic acid (SA) signaling, ISR is primarily associated with ET and jasmonic acid (JA) signaling pathways [56]. PGPR‐mediated ISR primes the plant immune system for faster and stronger defense activation following pathogen attack. These priming responses include reinforcement of plant cell walls, accumulation of antimicrobial secondary metabolites, and upregulation of pathogenesis‐related proteins [56]. Species such as *Bacillus subtilis* and *Pseudomonas fluorescens* have demonstrated strong abilities to activate these defense responses and improve plant resistance to diseases [56].

IAA produced by PGPR can enhance plant auxin signaling and stimulate root development. In addition, many PGPRs possess ACC deaminase activity, which breaks down ACC into ammonia and α‐ketobutyrate, thereby reducing stress ET levels in plants [57]. In nitrogen‐deficient soils, PGPRs such as *Bacillus* and *Pseudomonas* species, and symbiotic nitrogen‐fixing *Rhizobium* species, can enhance plant growth through multiple mechanisms including biological nitrogen fixation, nutrient solubilization, and phytohormone production [58]. Furthermore, improved plant development is also supported by biocontrol mechanisms such as the production of antibiotics and lytic enzymes that suppress phytopathogens [59]. Beyond direct phytopathogen inhibition, some PGPRs also indirectly enhance plant defense responses by improving plant vigor and stimulating the biosynthesis of defensive secondary metabolites. For example, AbdElgawad et al. [60] demonstrated that treatment of *Brassica oleracea* cultivars with *Nocardiopsis* species JM18 enhanced antibacterial activity through increased glucosinolate biosynthesis and activation of glucosinolate hydrolysis pathways. The hydrolysis of glucosinolates generates biologically active compounds, such as isothiocyanates, which possess strong antimicrobial properties [60].

PGPR application in agriculture enhances the eradication and protection of plants from infections by phytopathogenic microorganisms through the antagonistic compounds produced by PGPRs and ISR [61]. Collectively, these mechanisms demonstrate the important role of PGPRs in improving plant disease resistance and protecting medicinal and economically important plants against phytopathogenic infections.

#### 4.1.2. Stress Mitigation

Numerous biotic and abiotic stressors have a detrimental impact on plant health and productivity by producing ROS [62]. Plant growth can be hampered by abiotic factors such as temperature, salt stress, heavy metals, contaminated soil, flooding, and water deficit [63]. Therefore, ROS build up as a result of abiotic stress, which typically causes oxidative damage [62]. Furthermore, microorganisms enhance the growth of diverse crops grown under a range of stresses by preventing or reducing oxidative damage to plants through secretion of antioxidants [64]. Plant responses to abiotic stress are regulated by phytohormonal signaling networks and interactions with PGPRs, whereby PGPRs modulate hormone levels and signaling pathways to alleviate stress through both direct and indirect mechanisms [48].

Notably, plants have an antioxidant defense system and phenylpropanoid pathway to cope with biotic and abiotic stress [65]. The enzymes involved in the antioxidant defense system POX, SOD, CAT, APX, polyphenol oxidase (PPO), and phenylalanine ammonia‐lyase (PAL), as well as phenolic compounds, are crucial biomarkers that help in the assessment of defense against stress conditions [66]. PGPRs are crucial for reducing abiotic stress and activating the plant’s tolerance mechanism [67], as they mitigate the adverse effects of stresses by modulating plants’ antioxidant enzyme levels, especially SOD enzyme activity [68]. Thus, plants inoculated with beneficial microbes could increase the production of these enzymes and phenolic contents, enabling them to withstand biotic and abiotic stress [69]. Furthermore, the induction of ROS‐scavenging enzymes including SOD, POX, and CAT is the most common mechanism for detoxifying ROS synthesized under stress conditions [68]. Additionally, PGPR‐mediated physical or chemical changes in plants may also lead to increased plant tolerance against abiotic stresses, known as induced systemic tolerance (IST) [70]. For instance, inoculation with PGPRs can stimulate the activities of antioxidant enzymes and increase proline accumulation under salt stress conditions [68], thereby inducing systemic resistance against fungal plant diseases through the activation of various defense‐related enzymes. Improved antioxidative enzyme activity is often correlated with enhanced tolerance of plants to various abiotic stresses [71]. Also, SOD is a metalloenzyme, which constitutes an important element of the plant defense system against free radicals [70]. It catalyzes the conversion of superoxide radical (O_2_^*∙*−^) to hydrogen peroxide (H_2_O_2_) [72]. Furthermore, POX enzymes catalyze reactions in which hydrogen peroxide acts as an electron acceptor to oxidize various substrates, while APX catalyzes the reduction of H_2_O_2_ to water using ascorbate as an electron donor [70].

Research has demonstrated that stress‐tolerant bacteria can withstand the negative effects of harsh conditions by altering the morphology and structure of plant root systems, balancing oxidative and osmotic stress, and controlling ion homeostasis, among other mechanisms [73]. A variety of bacterial strains, including but not limited to *Bacillus amyloliquefaciens, Azospirillum brasilense, Rhizobium leguminosarum, and Mesorhizobium cicero*, have been reported to have strong resilience and promote plant growth under stress conditions [74]. Recent research has shown the role of PGPRs in modulating antioxidant enzyme levels in various crops in response to abiotic stresses, thereby reducing oxidative damage elicited by stress in plants [69].

In agreement with this, tomato plants treated with *Bacillus* strains, namely *B. subtilis* and *B. amyloliquefaciens*, resulted in increased activities of antioxidant machinery enzymes. *B. subtilis* strain IS1 and *B. amyloliquefaciens* strain IS6 showed increased activities of APX, CAT, POX, and SOD enzymes, which were attributed to improved plant protection against oxidative damage caused by salinity stress [75]. Moreover, in certain cases, PGPR‐treated plants tend to reduce antioxidant enzyme activities, indicating that the treated plants experienced less stress. In addition, Abdelshafy et al. [76] reported that inoculated tomato plants with *Bacillus sonorensis* (EGY05), *Bacillus tequilensis* (EGY21), and *Bacillus mojavensis* (EGY25) strains decreased (*p* < 0.05) the activity of antioxidant enzymes (SOD, CAT, and POX). However, the decline observed in the activities of antioxidant enzymes could be due to a general decrease in biosynthesis and lower oxidative stress.

#### 4.1.3. Growth Yield

PGPRs are one of the microorganisms that make up the soil microbiome [77]. They directly or indirectly promote plant growth and development by increasing the amount of mineral nutrients, controlling phytohormone levels, and bio‐controlling plant pathogens [71]. The phytohormones synthesized by PGPRs are auxins (e.g., IAA), cytokinins, gibberellins, and ET modulators, which control plant development and growth [78]. IAA is produced by strains such as *Azospirillum*, *Pseudomonas*, and *Bacillus*, and promotes root hair development, root elongation, and lateral root development [78]. For instance, *Bacillus amyloliquefaciens* strain TBorg1 culture filtrate (TBorg1‐CF) promoted tomato growth by significantly increasing the shoot and root lengths and the fresh weights of tobacco mosaic virus (TMV)–infected plants compared to TMV‐infected plants without CF treatments. These findings demonstrate the action of TBorg1 in IAA production, which may be responsible for the significant root and shoot growth observed in the TMV‐infected tomato plant [79]. In addition, gibberellin‐producing bacteria alter stem elongation and seed germination, while cytokinin‐producing PGPRs affect cell division and shoot development [80]. Also, some PGPRs can produce ACC deaminase, an enzyme that degrades 1‐ACC, the precursor of ET, a hormone that, when present in excess, inhibits plant growth under stress [81]. Thus, by reducing ET levels, PGPRs help plants retain growth under less‐than‐ideal conditions. For instance, Anand et al. [82] reported that an ACC deaminase–positive bacterial strain (*Enterobacter* strain K) enhanced plant growth and mitigated salinity stress in *Cajanus cajan* plants. The observed result can be attributed to the significant IAA production ability of strain K along with ACC deaminase activity, where the plant takes up the bacterially produced IAA and, together with endogenous IAA, counteracts the inhibitory effects of stress. Also, PGPRs change the physiology of the entire plant by increasing nutrient uptake and influencing the efficacy of root activities, resulting in improved plant yield [83]. It has also been reported that about 2%–5% of PGPRs improve the soil texture and plant yield [11].

#### 4.1.4. Enhancement of Secondary Metabolites

Various PGPR strains have been identified in association with medicinal plants and have been reported to enhance secondary metabolite accumulation and pharmacological properties [84]. Many secondary metabolites that are vital for human health are found in plant extracts or derivatives, which are the direct source of most medications [85]. Alkaloids, terpenoids, and phenolics are examples of secondary metabolites that can be enhanced with the application of PGPRs. These secondary metabolites contribute to enhanced tolerance to abiotic stresses and suppression of plant diseases.

PGPRs can synthesize secondary metabolites and influence their quantity and quality with their associated bioactivities [86]. For example, previous research findings have shown that *Pseudomonas*, *Bacillus*, and *Azospirillum* play a significant role in enhancing the biosynthesis of phenolic compounds, including flavonoids, in medicinal plants [87]. Also, several secondary metabolites are derived from microbial biosynthesis or from interactions between microbes and host plants [88]. Similarly, PGPRs have been reported to influence secondary metabolite production in medicinal plants, where inoculation is associated with enhanced secondary metabolite accumulation and activation of host plant immune responses [89].

### 4.2. The Influence of PGPRs on Various Secondary Metabolite Groups in Medicinal Plants

Secondary metabolites are plant chemicals with pharmacological properties that are capable of several bioactivities [90]. They have a variety of pharmacological properties, such as, but not limited to, antioxidant, anti‐inflammatory, anticancer, antiallergic, antidiabetic, and antibacterial [91]. In addition to being beneficial for human health, these secondary metabolites shield plants from biotic and abiotic stress [92]. Secondary metabolites can be classified based on their chemical composition. They are divided into three broad categories: alkaloids, phenolics, and terpenoids [8].

The synthesis of plant secondary metabolites is governed by complex and highly regulated biochemical pathways [93]. PGPRs can modulate these pathways through multiple mechanisms, including the production of phytohormones, elicitors, and VOCs, as well as other signaling molecules [94]. These bacterial signals influence plant regulatory networks by activating specific transcription factors, thereby altering the expression of biosynthetic genes involved in secondary metabolism [95].

PGPR‐mediated enhancement of the bioactivities of medicinal plants is primarily attributed to their ability to modulate plant secondary metabolism rather than exerting direct pharmacological effects [94]. Upon colonization of the rhizosphere or plant tissues, PGPRs activate plant signaling networks that involve JA, SA, ET, abscisic acid (ABA), and other defense‐related pathways [89] (Figure 2). These signaling events upregulate key biosynthetic enzymes such as PAL, chalcone synthase (CHS), tyrosine aminotransferase (TAT), and strictosidine synthase (STR), as well as metabolic pathways responsible for the production of secondary metabolites, including phenolics, flavonoids, terpenoids, alkaloids, and glycosides [95]. As these secondary metabolites accumulate, the medicinal plant extracts often exhibit enhanced antioxidant, antimicrobial, anti‐inflammatory, antidiabetic, or anticancer activities [91]. However, enhanced bioactivities are influenced not only by increased levels of secondary metabolites but also by qualitative changes in their chemical composition and metabolite profiles resulting from PGPR‐mediated modulation of plant secondary metabolism [69], thus emphasizing the necessity of validating secondary metabolite changes with appropriate bioactivity assays.

**FIGURE 2:**
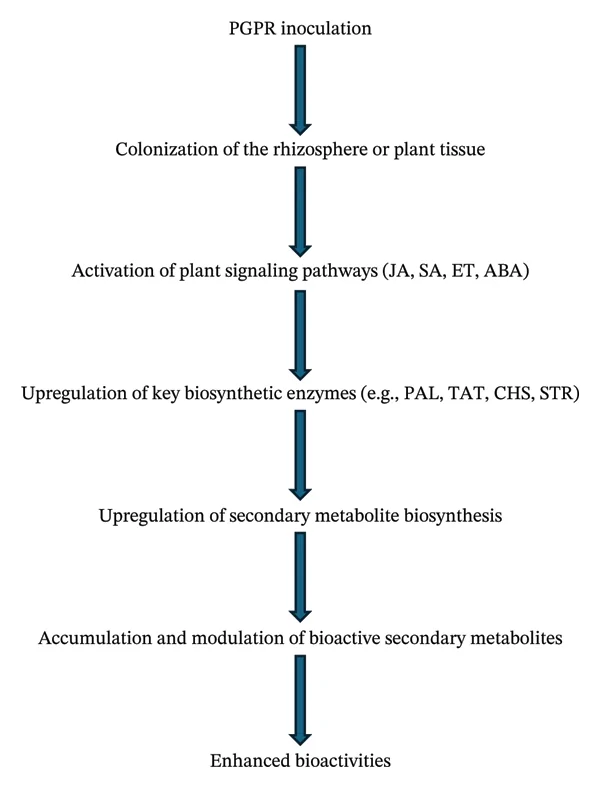
Proposed mechanistic pathway linking PGPR inoculation to enhanced bioactivities in medicinal plants.

Among the most extensively studied metabolite groups influenced by PGPRs are phenolic compounds, including lignins, tannins, and flavonoids [69]. In many plant–PGPR interactions, inoculation has been associated with enhanced activity of the phenylpropanoid pathway, which is central to the biosynthesis of these compounds; however, the magnitude and direction of this response are dependent on plant species, bacterial strain, and environmental conditions [96].

Lately, research has demonstrated that PGPRs have a major impact on the synthesis of secondary metabolites in medicinal plants. The rapid advancement of high‐throughput sequencing technologies has revealed a strong relationship between PGPRs and the accumulation of secondary metabolites in medicinal plants [97]. Table 2 shows the different secondary metabolites enhanced with the application of PGPRs in specific medicinal plants based on the selected articles used in this review.

**TABLE 2:** Secondary metabolites improved with the application of PGPRs in various medicinal plants from the literature search.

S/n	Host plants	Type of PGPR strains/consortia	Inoculation method	Experimental details	Secondary metabolites	Bioactivity	Experimental location	Reference
1	*Mentha pulegium* L.	*Azotobacter chroococcum* and *Azospirillum brasilense* (Rhizospheric)	Soil drench	Greenhouse	Flavonoids, phenolics, monoterpenes	Antioxidant	Iran, Asia	[98]
2	*Mentha piperita*	*Pseudomonas putida* SJ04 and *Bacillus amyloliquefaciens* GB03 (Rhizospheric)	Soil drench	Growth chamber	Essential oil, phenolic compounds	Not indicated	Argentina, South America	[99]
3	*Coptis teeta*	*Microbacterium* and *norank_f_*7B8, (Endophytic)	Root dipping	Natural and artificial cultivation	Berberine	Not indicated	China, Asia	[100]
4	*Lessertia frutescens* (L.)	*Rhizobium* strain (Rhizospheric)	Soil drench	Field	Phenolics, flavonoids	Antioxidant	South Africa, Africa	[101]
5	*Artemisia annua*	*Bacillus subtilis, B. licheniformis, Burkholderia* sp., and *Acinetobacter pittii* (Endophytic)	Root dipping	Glasshouse	Artemisinin	Not indicated	India, Asia	[102]
6	*Anoectochilus roxburghii* (Wall.) *Lindl.* and *Anoectochilus formosanus* (Wall.)	*Bacillus velezensis* D2WM (D2) and *Bacillus velezensis* ZJ‐11 (ZJ‐11), (Rhizospheric)	Rhizosphere	Greenhouse	Flavonoids	Not indicated	China, Asia	[103]
7	*Polygonum cuspidatum*	*Bacteroides* sp*., Acinetobacter* sp*., Erysipelatoclostridium* sp., and *Achromobacter* sp*.* (Rhizospheric)	Bulk and rhizosphere soil	Field	Resveratrol	Not indicated	China, Asia	[104]
8	*Thymus vulgaris* L.	*Bacillus haynesii *T9r*, Citrobacter farmeri* T10r*, Bacillus licheniformis* T11r*, Bacillus velezensis* T12r, and *Bacillus velezensis* T13r*.* (Endophytic)	Root	Field	Essential oil	Not indicated	Egypt, Africa	[105]
9	*Sophora flavescens*	Actinobacteria and Chloroflexi (Rhizospheric)	Rhizosphere and root	Field	Matrine and oxymatrine	Not indicated	China, Asia	[106]
10	*Solanum lycopersicum*	*Bacillus *spp. (5PB1, 1PB1, FV46) and *Brevibacillus *sp. (C9F) (Rhizospheric)	Seed soaking and soil	Greenhouse	Phenol	Antioxidant	Portugal, Europe	[107]
11	*Cichorium endivia* L.	*Rhizobium laguerreae* SCCPVE07 and *Bacillus halotolerans* PEPV40 (Rhizospheric)	Root dipping	Growth chamber	Protocatechuic acid, caffeic acid, or kaempferol 3‐*O*‐glucuronide	Not indicated	Spain, Europe	[108]
12	*Panax ginseng*	*Rhizobium panacihumi* DCY116^T^ (Rhizospheric)	Seed dipping	Pot	Phenolics	Antioxidant	Korea, Asia	[109]
13	*Ocimum basilicum, Satureja hortensis*	*Sinorhizobium meliloti* Rm1021, *Bacillus subtilis* BS119 m, *Bradyrhizobium* sp. USDA 4438 and *Pseudomonas fluorescens* WCS417 r.+ *Glomus intraradices* and *G. mosseae* (Rhizospheric)	Seed coating	Field	Essential oil	Not indicated	Iran, Asia	[47]
14	*Astragalus mongholicus*	*Stenotrophomonas *sp. *Phyllobacterium* sp*. and Inquilinus* sp. (Rhizospheric)	Rhizosphere and root	Field	Astragaloside, Calycosin	Not indicated	China, Asia	[110]
15	*Allium cepa *L.	*Azospirillum brasilense, Gluconacetobacter diazotrophicus, Herbaspirillum seropedicae, and Burkholderia ambifaria* (Endophytic)	Seed immersion	Field	Phenolics	Antioxidant	Italy, Europe	[111]
16	*Brassica napus *(canola)	*Azospirillum brasilense*, *Arthrobacter globiformis*, *Burkholderia ambifaria Herbaspirillum seropedicae*, and *Pseudomonas* sp. (Rhizospheric)	Seed inoculation after sowing	Pot experiment in natural environment	Phenolic compounds	Antioxidant	Italy, Europe	[112]
17	*Fraxinus excelsior*	*Paenibacillus *sp. and *Pseudomonas *sp. (Endophytic)	Spot inoculation with inoculation needle	Petri dish with Murashige and Skoog (MS) medium	Phenol, flavonoid, and carotenoid	Not indicated	Lithuania, Europe	[113]
18	*Alkanna tinctoria *L.	*Labrys*, *Allorhizobium–Neorhizobium–Pararhizobium–Rhizobium*, and *Penicillium* sp. (Rhizospheric)	Soil inoculation	Greenhouse	Alkannins	Not indicated	Greece, Europe	[114]
19	*Melissa officinalis* L.	*Azospirillum brasilense* (Sp245) strain) *+ Glomus mosseae* (Rhizospheric)	Seed soaking	Greenhouse	Essential oil	Not indicated	Iran, Asia	[115]
20	*Arabidopsis* sp.	*Aeromonas *sp. H1, *Bacillus amyloliquefaciens* GB03, *B. megaterium* YCR‐R4, and *B. megaterium* TG1‐E1 (Rhizospheric)	Soil inoculation	Growth chamber	Flavonoids	Not indicated	China, Asia	[116]
21	*Astragalus mongholicus*	Bacterial consortium 6—The isolate HQB9, HQB56, HQB13, HQB383, HQB90, and HQB216 belong to *Bacillus*, *Acinetobacter*, *Leclercia*, *Sphingomonas*, *Microbacterium*, and *Brevibacterium* (Rhizospheric)	Soil inoculation	Glasshouse	Astragaloside IV, calycosin‐7‐glucoside	Not indicated	China, Asia	[117]
22	*Lycium barbarum*	*norank_o_Gaiellales* and *norank_f_Anaerolineaceae* AKYG1722, which are classified as Chloroflexi and Actinobacteria (Rhizospheric)	Rhizosphere soil	Field	Flavonoids	Not indicated	China, Asia	[118]
23	*Origanum vulgare subsp. vulgare*	*Bacillus *strains *Arthrobacter* and *Paenibacillus* strains (Endophytic)	Not indicated	Not indicated	VOCs, monoterpenes	Antimicrobial	Italy, Europe	[119]
24	*Adhatoda vasica*	*Azotobacter chroococcum* and *Pseudomonas putida* (Rhizospheric)	Seeds were inoculated onto semisolid Murashige and Skoog (MS) culture medium	Hairy root development from leaf discs grown on nutrient agar medium.	Pyrroloquinazoline alkaloids	Not indicated	India, Asia	[120]
25	*Helianthus annuus*	*Ralstonia pickettii* and *Brevibacillus invocatus* (Rhizospheric)	Seed soaking	Greenhouse	Flavonoids, phenolics	Not indicated	USA, North America	[50]
26	*Curcuma longa*	*Serratia nematodiphila* RGK, *Pseudomonas plecoglossicida* RGK (Rhizospheric)	Rhizomes soaking/dipping	Pot culture experiment in natural environment	Phenolic, flavonoids	Antioxidant, antifungal	India, Asia	[121]
27	*Glycine max*	*Acinetobacter pittii *YNA40 (Rhizospheric)	Soil drench	Growth chamber	Flavonoids, phenolics	Antioxidant	Korea, Asia	[122]
28	*Glycyrrhiza uralensis*	*Rhizobium rhizolycopersici* GUH2 (Endophytic)	Soil drench	Pot experiment in controlled environment	Isoliquiritin and glycyrrhizic acid	Not indicated	China, Asia	[123]
29	*Capsicum annuum*	*Pseudomonas *42P4*, Cellulosimicrobium* 60I1, *Ochrobactrum* 53F, and *Enterobacter* 64S1 (Rhizospheric)	Root dipping	Greenhouse	Carotenoid, phenolic, and flavonoid compounds	Not indicated	Argentina, South America	[87]
30	*Cannabis sativa* L.	*Bacillus* sp., *Mucilaginibacter* sp., and *Pseudomonas* sp. (Rhizospheric)	Soil drenching	Growth room	Terpene, cannabinoids	Not indicated	Canada, North America	[124]
31	*Cucumis sativus* L.	*Bacillus cereus *(KBEndo4P6), *Acinetobacter radioresistens* (KBEndo3P1), *Pseudomonas paralactis* (KBEndo6P7), *and Sinorhizobiu meliloti* (KBEkto9P6) (Rhizospheric)	Seed priming	Growth chamber	Phenolic compounds	Antioxidant	Mexico, North America	[125]
32	*Origanum vulgare* L.	*Arthrobacter *sp. OVS8 (Endophytic)	Not indicated	Garden	VOCs	Antimicrobial	Italy, Europe	[126]
33	*Brassica juncea *L.	*Pseudomonas aeruginosa, Burkholderia gladioli + Eisenia fetida* (Rhizospheric)	Soil drenching	Pot experiment in natural condition	Total phenols, flavonoids	Not indicated	India, Asia	[59]
34	*Allium sativum* L.	*Pseudomonas putida* (KX574857), *Pseudomonas stutzeri* (KX574858), and *Bacillus cereus* (ATCC14579) (Rhizospheric)	Cloves soaking	Net house under natural conditions	Flavonoids, phenolics	Not indicated	Pakistan, Asia	[127]
35	*Salicornia europaea *L.	*Brevibacterium casei *EB3 *and Pseudomonas oryzihabitans *RL18 (Rhizoendophytic)	Seed immersion	Controlled laboratory and field experiment	Phenolic compounds; hydroxybenzoic acid, quercetin, and apigenin	Not indicated	Portugal, Europe	[128]
36	*Medicago truncatula*	*Pseudomonas brassicacearum *KK5, *P. corrugata* KK7*, Paenibacillus borealis* KK4, and *Sinorhizobium meliloti* KK13 (Rhizospheric)	Soil inoculation with pipette near the root system	Controlled environment	Phenolic compounds	Not indicated	Poland, Europe	[129]
37	*Vicia faba *L.	*Cellulomonas pakistanensis *(National Culture Collection of Pakistan [NCCP] 11), and *Sphingobacterium pakistanensis* (NCCP246) (Rhizospheric)	Seed soaking	Controlled‐pot experiment	Flavonoids, phenols	Not indicated	Pakistan, Asia	[130]
38	*Pseudostellaria heterophylla*	*Bacillus subtilis *and *Pseudomonas fluorescens* (Rhizospheric)	Soil drenching	Climate‐controlled chamber	Alkaloids	Not indicated	China, Asia	[131]
39	*Sesamum indicum *L.	*Pseudomonas toyotomiensis*, *Bacillus subtilis*, and *Bacillus cereus* (Rhizospheric)	Soil drenching	Greenhouse	Flavonoids	Antioxidant	India, Asia	[132]
40	*Brassica oleracea*	*Nocardiopsis *sp. (JM18) (Rhizospheric) + selenium nanoparticles (SeNPs)	Seed immersion	Growth chamber	Total phenolic and total flavonoid	Antibacterial, Antioxidant	Egypt, Africa	[60]
41	*Solanum lycopersicum*	*Bacillus amyloliquefaciens *strain TBorg1 (Rhizospheric)	Foliar application	Greenhouse	Phenol	Antioxidant, Antiviral	Egypt, Africa	[79]
42	*Lycopersicon esculentum *Mill.	*Bacillus endophyticus* and *Pseudomonas aeruginosa* (Rhizospheric)	Seed soaking	Greenhouse	Flavonoids	Not indicated	Islamabad, Asia	[133]
43	*Glycyrrhiza uralensis *F.	*Bacillus amyloliquefaciens *FZB42 (Rhizospheric)	Rhizosphere	Greenhouse	Flavonoid—liquiritin and glycyrrhizic acid	Not indicated	China, Asia	[134]
44	*Cicer arietinum *L.	*Azospirillum brasilense—*EMCC1454 (Rhizospheric)	Root dipping	Growth chamber	Phenolics, flavonoids	Antioxidant	Egypt, Africa	[135]
45	*Ocimum sanctum*	*Trichoderma harzianum+* *Halomonas desiderata, Bacillus subtilis, Achromobacter xylosoxidans, Burkholderia cepacia, Brevibacterium halotolerans* (Rhizospheric)	Root dipping and soil drenching	Glasshouse	Phenol, flavonoids	Not indicated	India, Asia	[136]
46	*Citrus reticulata*	*Streptomyces* strain Strep‐4 (Rhizospheric)	Soil drenching/root dipping	Field	Monoterpene	Not indicated	China, Asia	[137]

#### 4.2.1. Alkaloids

Alkaloids are organic heterocyclic compounds with at least one nitrogen atom either inside or outside the ring [138]. They are produced from amino acids, with their specific bioactivity depending on their chemical structure [139]. Furthermore, they are involved in the germination of plants and defense against herbivory and harmful microorganisms [139]. Alkaloids have several pharmacological properties, such as analgesic, anticancer, antibacterial, antimalarial, anti‐inflammatory, and antiarrhythmic.

The application of PGPR strains plays a significant role in expediting the biosynthesis of alkaloids in medicinal plants [89]. Studies have reported changes in plant alkaloid content following inoculation with diverse PGPR strains [100]. For instance, Chen et al. [106] investigated the effects of the association of the rhizosphere bacterial microbiota on the accumulation of alkaloids in the roots of *Sophora flavescens*. The results indicated an increase in the alkaloid content mostly due to the effect of Actinobacteria and Chloroflexi, highlighting the beneficial effects of these bacterial consortia on the biochemical quality of the plant. Similar results were reported by Singh et al. [120], where *Azotobacter chroococcum* and *Pseudomonas putida* significantly enhanced the pyrroloquinazoline alkaloid accumulation in *Adhatoda vasica.* Most recently*, Bacillus subtilis and Pseudomonas fluorescens* were reported to improve the production of alkaloids in *Pseudostellaria heterophylla* under ambient CO_2_ and eCO_2_ conditions, respectively [131]. The observed results in these studies may be attributed to plant hormones such as JA and SA, which modulate the expression and activity of alkaloid biosynthetic enzymes, including tryptophan decarboxylase (TDC) and STR [140].

PGPRs are reported to influence alkaloid biosynthesis via modulation of phytohormone signaling pathways, particularly under stress conditions [141]. This regulation often involves the upregulation of key biosynthetic enzymes, such as tropinone reductase or STR, depending on the plant species. Among these signaling molecules, JA is especially recognized for activating alkaloid biosynthetic pathways and enhancing the expression of enzymes involved in secondary metabolite production [142].

#### 4.2.2. Flavonoids

Flavonoids are a broad polyphenolic class of chemicals and secondary metabolites found naturally in plants [143]. Flavones, isoflavones, flavonols, flavanonols, flavanones, flavanols or catechins, anthocyanins, and chalcones are some of the subgroups of flavonoids. Flavonoids are valuable therapeutic compounds due to their high antioxidant, anti‐inflammatory, antiviral, and anticancer effects. They are antioxidants and perform multiple functions in plants [143].

Several studies reported increased production of phenolic compounds following PGPR application [98, 116, 117, 121, 123, 130, 132]. Likewise, Makgato et al. [101] reported that rhizobium inoculation improved the content of flavonoids in *Lessertia frutescens* (L.) with increased antioxidant activity. Similarly, Wei et al. [103] found that *Anoectochilus* plants had a 44.70% increase in flavonoid content when inoculated with two *Bacillus velezensis* strains. Furthermore, Striganavičiūtė et al. [113] highlighted the potential of *Paenibacillus* sp. and *Pseudomonas* sp., which significantly increased the total flavonoid content in European ash (*Fraxinus excelsior* L.). Notably, PGPRs are known to increase the production of phenolic compounds, including flavonoids, through the activation of the phenylpropanoid pathway [69]. In particular, plants treated with beneficial bacteria frequently show increased expression or activity of PAL, a key enzyme involved in flavonoid biosynthesis. In another study, bacterial strains of *Ralstonia pickettii* and *Brevibacillus invocatus* also played a significant role in the improvement of the flavonoid content in the inoculated sunflower plants. The flavonoid content was recorded to be 0.54 times higher in *Ralstonia pickettii*–inoculated plants [50]. In similar studies, Lui et al. [118] reported that *norank_o_Gaiellales* and *norank_f_*AKYG1722, classified under the phyla Actinobacteria and Chloroflexi, respectively, significantly enhanced flavonoid accumulation in *Lycium barbarum*. More recently, inoculation of soybean with *Acinetobacter pittii* YNA40 was also shown to improve flavonoid content and diphenyl‐1‐picrylhydrazyl (DPPH) radical scavenging activity [122]. The effects observed in these studies may be associated with increased PAL activity, which plays a central role in the phenylpropanoid pathway involved in flavonoid biosynthesis.

#### 4.2.3. Phenolic Compounds

Phenolic compounds are secondary metabolites with one or more phenol groups [129], and these secondary metabolites are highly valued for their pharmacological properties. Phenolic compounds comprise a diverse group of secondary metabolites, including simple phenols, tannins, coumarins, flavonoids, lignans, stilbenes, and xanthones [144].

PGPRs have a significant influence on phenolic compounds, which have been the subject of considerable research. Typically, PGPR inoculation stimulates the phenylpropanoid pathway, leading to the production of these secondary metabolites [11]. The mechanism of action of PGPRs involves enhancement of the expression and activity of PAL, a key enzyme involved in the biosynthesis of phenolic compounds [129]. For instance, several studies on PGPR strains and their effect on the accumulation of phenolic compounds in medicinal plants were reported by Asghari et al. [98], Makgato et al. [101], Zhang et al. [104], Costa‐Santos et al. [107], Jiménez‐Gómez et al. [108], Li et al. [110], Pellegrini et al. [111], Rossi et al. [112], Striganaviciute et al. [113], Csorba et al. [114], Urooj et al. [50], Kang et al. [122], Sharma et al. [59], Tariq et al. [127], Nafees et al. [130], and Kisiel et al. [129]. Furthermore, Kang et al. [109] reported increased phenolic compound accumulation, as well as activation of the antioxidant system, in *Panax ginseng* seedlings treated with *Rhizobium panacihumi* DCY116T. Also, *Acinetobacter radioresistens* (KBEndo3P1) was reported to improve the total phenolic content in *Cucumis sativus* [125]. Another study by Lobato‐Ureche et al. [87] evaluated the effects of *Pseudomonas* 42P4 and *Cellulosimicrobium* 60I1 strains on pepper plants, and the results showed improvements in the phenolic compound profile. In addition, Jagtap et al. [121] reported, for the first time, the presence of the phenolic compound 4‐hydroxy‐2‐methylacetophenone in consortium‐treated turmeric plants, which exhibited significant antioxidant activity. Lastly, Ferreira et al. [128] showed that inoculation with *Brevibacterium casei* EB3 and *Pseudomonas oryzihabitans* RL18 improved the phenolic compound content of *Salicornia europaea* L. These findings may be attributed to the ability of PGPRs to induce plant defense responses and secondary metabolism, thereby enhancing PAL activity and increasing the production of phenolic compounds.

#### 4.2.4. Terpenoids

Terpenoids, also known as isoprenoids, are a large class of naturally occurring organic compounds derived from 5‐carbon isoprene units. This class includes terpenes, namely hydrocarbons and terpenoids, which are modified terpene compounds containing additional functional groups [139]. Their medicinal, antibacterial, and antioxidant properties make them highly important in the culinary, cosmetic, and pharmaceutical industries.

Reports by Striganaviciute et al. [113] demonstrated the improved effects of *Paenibacillus* sp. and *Pseudomonas* sp. on the carotenoid content of European ash (*Fraxinus excelsior*). Similarly, Lyu et al. [124] investigated the effects of coinoculation with *Mucilaginibacter* sp. and *Pseudomonas* sp. in *Cannabis sativa* L. Their results showed that coinoculation increased the total terpene content by 23% and 18%, respectively. Several other studies have also reported the positive influence of PGPR strains on terpenoid content in various medicinal plants [102, 123, 145].

These findings may be associated with the involvement of the mevalonate (MVA) and methylerythritol phosphate (MEP) pathways, which play crucial roles in plant terpenoid biosynthesis. PGPR‐induced activation of these pathways may enhance the synthesis of terpenoid‐derived compounds, including carotenoids, certain saponins, and essential oils. Research has shown that bacterial VOCs, such as acetoin and 2,3‐butanediol, can modulate the expression of genes involved in the MVA and MEP pathways, thereby stimulating terpenoid biosynthesis [146].

#### 4.2.5. VOCs

VOCs emitted by plants are low‐molecular‐weight carbon‐based compounds with high vapor pressures and low boiling points, allowing them to evaporate easily under ambient conditions [147]. These compounds also possess diverse bioactivities, including antibacterial, anti‐inflammatory, and antioxidant properties. In plant–microbe interactions, root‐emitted VOCs serve as signaling molecules that mediate communication between plants and microorganisms. VOCs can enhance plant growth and health by attracting beneficial microbes and suppressing harmful organisms [147].

PGPRs influence plant VOC emissions indirectly through ISR (stress priming), enhanced nutrient uptake, and the elicitation of plant defense signaling pathways, which together modulate plant metabolism and volatile profiles [148]. For instance, according to a study by Cappellari and colleagues, an increase in the essential oil content of peppermint (*Mentha *×* piperita* L.) was observed when plants were inoculated with *Pseudomonas putida* SJ04 or *Bacillus amyloliquefaciens* GB03. The results showed a 3.5‐fold increase in essential oil content and a 3‐fold increase in other VOCs compared with the control samples [99].

Furthermore, Polito et al. [119] showed VOC accumulation in response to endophytic bacteria associated with *Origanum vulgare* spp. The accumulated VOCs exhibited strong antimicrobial properties. Similarly, *Arthrobacter* sp. OVS8 modified the VOC profile in *Origanum* species in *Origanum vulgare* L. [126]. In addition, improvements in sesame oil quality and antioxidant activity were observed in *Sesamum indicum* L. plants following inoculation with *Pseudomonas toyotomiensis*, *Bacillus subtilis*, and *Bacillus cereus* [132]. Other studies have also reported the beneficial effects of PGPRs on VOC content in medicinal plants [47, 98, 105, 115].

## 5. Research Gaps, Limitations, and Recommendations Based on the Reviewed Literature

This review aimed to provide information on recent studies regarding the beneficial roles of PGPRs in medicinal plants, specifically focusing on the accumulation of secondary metabolites (Table 2). Although this topic has been relatively well studied, several gaps remain, including the lack of standardized inoculation methods, limited controlled‐environment studies, variability in PGPRs’ performance, and a limited understanding of the relationship between PGPR‐induced secondary metabolites and their bioactivity.

The application of PGPRs in medicinal plant production is still not fully understood. Most studies report increases in metabolite concentrations without evaluating the associated bioactivities, while the underlying mechanisms responsible for these changes remain unclear. It is still unknown whether PGPRs directly activate biosynthetic pathways or whether the observed effects are secondary consequences of improved plant health and nutrient availability. Significant knowledge gaps therefore exist regarding the influence of PGPRs on metabolic pathways in medicinal plants. This mechanistic ambiguity limits the ability to predict and optimize PGPRs’ performance in medicinal plant production systems. Consequently, these inconsistencies and unresolved mechanisms hinder the wide adoption of PGPR technologies.

### 5.1. Limitations of Controlled‐Environment Studies

A major concern in PGPR research is heavy reliance on greenhouse and laboratory studies (Table 2). Such controlled environments allow for the separation of specific variables and in‐depth study of plant–microbe interactions that in fact do not accurately mimic real‐world agricultural settings [149]. Furthermore, most studies focus on the response of plants to a single abiotic factor, while in nature, plants experience stress caused by a combination of abiotic factors [150]. Therefore, more field‐based research trials should be incorporated. Incorporating more field‐based research will provide a more accurate assessment of the effects of PGPR application in agricultural practices, and this will help determine real‐world feasibility and economic viability [149].

Field environments are inherently complex with many interacting factors such as soil heterogeneity, climatic variability, and microbial competition [151]. These factors can significantly influence the survival, colonization, and activity of PGPRs. Thus, the results of controlled studies frequently do not necessarily translate to field applications [151]. However, multistress research incorporating various factors within a controlled environment can provide a point of departure for field experiments. Furthermore, many studies undertaken in controlled environments are short‐term, focusing on early plant growth stages rather than long‐term productivity and sustainability. This gap limits the understanding of the persistence and long‐term effects of PGPRs in agricultural systems. There is a clear need for multiseason field trials evaluating PGPR performance in a range of environmental conditions, particularly in regions where medicinal plants are extensively cultivated.

### 5.2. Research Gap in PGPR Inoculation Methods and Secondary Metabolite Enhancement in Medicinal Plants

Existing studies have demonstrated the important role of PGPRs in improving the growth, productivity, and accumulation of secondary metabolites in medicinal plants (Table 2). Recent research has highlighted the potential of PGPRs to enhance the synthesis of valuable secondary metabolites such as alkaloids, phenolics, flavonoids, and terpenoids, which are essential for the medicinal value of these plants. However, despite the increasing number of studies in this field, one major limitation is the lack of methodological standardization across studies. Different studies use different PGPR inoculation methods [152], including seed treatment, soil application, root dipping, and foliar spraying, often without clear justification for the selected approach. Variations in inoculum concentration, frequency of application, environmental conditions, and plant developmental stages further contribute to inconsistencies in research outcomes [153]. As a result, it becomes difficult to compare findings across studies or determine the most effective inoculation strategy for enhancing secondary metabolite accumulation in medicinal plants.

Additionally, many studies focus primarily on plant growth promotion rather than providing detailed mechanistic explanations of how PGPRs influence secondary metabolite biosynthetic pathways. There is also limited information regarding the long‐term effectiveness of PGPR inoculation under field conditions compared to controlled greenhouse experiments. Furthermore, the interaction between specific PGPR strains and different medicinal plant species remains insufficiently explored, particularly under concurrent abiotic stress conditions such as drought, salinity, and temperature fluctuations.

Therefore, further research is needed to establish standardized PGPR inoculation protocols and to investigate the mechanisms through which PGPRs regulate secondary metabolite production in medicinal plants. Such studies would improve reproducibility, facilitate comparison among findings, and support the development of sustainable agricultural and pharmaceutical applications.

### 5.3. Variability and Inconsistency in the Performance of PGPRs

One of the main challenges in PGPR research is the inconsistency of results across studies [154]. Some reports show dramatic increases in plant growth and production of metabolites, while others show little to no effect. This variation can be attributed to several factors such as the different bacterial strains, plant species, soil conditions, and experimental design [154]. The effects of PGPRs are often strain‐specific, meaning that a strain that is effective in one context may not perform well in another. Moreover, medicinal plants show a range of physiological and biochemical characteristics that could affect their response to PGPR inoculation. These characteristics include differences in root architecture, nutrient uptake efficiency, secondary metabolite production, phytohormone regulation, and root exudate composition, all of which may influence PGPR’s colonization and activity. Variations in these plant traits may therefore contribute to inconsistent PGPR performance among different medicinal plant species and environmental conditions. Another factor responsible for this is host specificity [155]. Plants contain receptors (PRRs) that detect specific molecules from microbes. Since different plants have different receptors, they recognize PGPR strains differently [154]. Plants that recognize certain PGPR’s molecules more effectively may show stronger beneficial growth responses. The impact of these factors should be considered to prevent generalized conclusions that might not be applicable to all hosts.

Notably, some studies only used single‐strain inoculations. Plants in natural soil ecosystems are therefore exposed to complex microbial communities rather than isolated strains. Therefore, the use of single strains can oversimplify plant–microbe interactions and underestimate the synergistic effects of microbial consortia [156]. Therefore, microbiome engineering is a promising future direction for PGPR application in medicinal plants by enabling the deliberate manipulation of plant‐associated microbial communities to enhance beneficial plant–microbe interactions. Unlike the application of single PGPR strains, microbiome engineering aims to establish stable and synergistic microbial consortia that improve plant growth, stress tolerance, secondary metabolite biosynthesis, and the bioactivities of medicinal plants under diverse environmental conditions. Further studies on multistrain formulations in medicinal plant research should be conducted, as this would help facilitate the development of more reliable and effective biostimulants.

### 5.4. Limited Understanding of the Relationship Between PGPR‐Induced Secondary Metabolites and Bioactivity

Although numerous studies have reported that plant PGPRs enhance the production of secondary metabolites in plants, there remains limited research investigating the direct association between PGPR‐induced metabolite enhancement and the resulting bioactivity or efficacy of the plant extracts (Table 2). Most existing studies primarily focus on increases in metabolite concentration without comprehensively determining whether these increases translate into improved bioactivity, such as antioxidant, antimicrobial, anti‐inflammatory, or therapeutic effects (Figure 3). Consequently, the relationship between enhanced metabolite accumulation and functional bioactivity remains insufficiently understood [157]. Analysis of the reviewed studies published between 2020 and 2025 indicates that approximately 73.4% reported quantitative increases in secondary metabolite concentrations or essential oil yields without evaluating corresponding biological activities. Only 26.6% assessed whether these phytochemical changes were associated with biological activity using functional in vitro or in vivo assays, such as DPPH or ABTS radical scavenging assays, antimicrobial minimum inhibitory concentration (MIC) assays, or anti‐inflammatory biomarkers (Figure 3).

**FIGURE 3:**
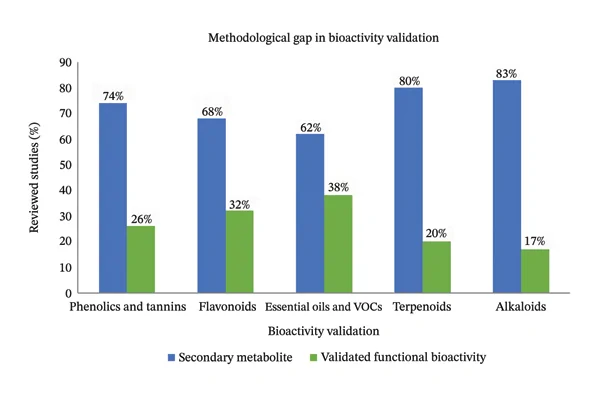
Comparison of studies reporting secondary metabolite accumulation alone and those validating functional bioactivity.

This decoupling is especially pronounced in alkaloid (83% unvalidated) and terpenoid research (80% unvalidated), whereas phenolic and essential oil studies exhibit higher rates of functional verification. Increased secondary metabolite concentrations do not necessarily result in proportional increases in biological activity, as the overall effect also depends on the chemical composition of the metabolites and potential interactions among individual bioactive compounds. Addressing this research gap is essential because it would establish whether PGPR‐mediated increases in secondary metabolites genuinely contribute to enhanced biological efficacy. Such studies would also provide a clearer understanding of the mechanisms through which PGPRs influence plant secondary metabolism and bioactivity, thereby supporting the development of more effective and scientifically validated PGPR‐based strategies for improving the medicinal and functional value of plants.

Therefore, future research should integrate metabolomics with bioactivity assays to identify the specific secondary metabolites and metabolic pathways responsible for enhanced bioactivities following PGPR inoculation. By linking metabolite profiles with experimentally validated bioactivity data, this integrated approach would provide mechanistic insights into how PGPR‐induced modulation of secondary metabolism contributes to the pharmacological potential of medicinal plants.

### 5.5. Molecular and Biochemical Knowledge Gaps

Progress in molecular biology has provided powerful tools to study plant–microbe interactions [18]. Genomics, transcriptomics, and metabolomics approaches have the potential to reveal the molecular mechanisms behind the effects of PGPRs [158]. However, the application of these techniques to PGPR studies related to medicinal plants is still very limited. Most of the studies rely on phenotypic observations such as increased biomass or metabolite concentration without probing the genetic or biochemical pathways involved. Limited molecular knowledge limits the development of targeted and efficient PGPR applications [159]. Moreover, the interaction between PGPRs and plant secondary metabolism remains understudied. Secondary metabolites are often produced in response to environmental stress and signaling pathways, yet the mechanisms through which PGPRs influence these processes remain unclear. Understanding these interactions is crucial for optimizing the use of PGPRs to enhance the quality of medicinal plants.

### 5.6. Geographical and Contextual Research Gaps

Another critical limitation in the current literature is the geographical focus of PGPR studies. Although many studies have been conducted in Asia, America, and Europe, few investigations have been conducted in African regions (Table 2). This is a major concern considering the abundance of medicinal plants in Africa and the importance of traditional medicine to local communities. There are marked regional differences in environmental factors such as soil type, climate, and native microbial populations [160]. Therefore, PGPR strains developed in one region may not perform well in another [161]. The dearth of research in the region constrains the generalizability of present findings and underscores the necessity for localized research. South Africa, with a high diversity of medicinal plants and variable agricultural conditions [162], clearly needs research into the indigenous plant species and local environmental conditions. These studies would provide valuable insights into the development of regionally adapted PGPR technologies.

### 5.7. Practical and Commercialization Challenges

The commercial application of PGPR in medicinal plant cultivation remains limited due to several interconnected challenges. Several practical challenges, including regulatory approval pathways, formulation technologies, limited shelf life of microbial products, variability in field performance, quality control, commercialization of microbial inoculants, farmer adoption, and economic feasibility, hamper their widespread use [163].

**Regulatory approval pathways** are often lengthy and differ between countries, delaying the registration and market entry of microbial inoculants. Although commercial biostimulant products are available, their quality and effectiveness vary considerably. This inconsistency affects farmers’ confidence and highlights the need for stronger regulatory frameworks and standardized quality‐control measures. Furthermore, regulatory approval processes for the commercialization of PGPRs should be streamlined and made more cost‐effective [163]. **Quality control** is another critical concern. This is essential for the commercialization of PGPR‐based products in medicinal plant cultivation, as standardized manufacturing protocols are required to ensure microbial purity, accurate strain identity, high bacterial viability, and consistent efficacy across production batches [164]. These measures are necessary to guarantee product reliability, reproducibility, and farmer confidence under field conditions. Also, the development of effective **formulation technologies** remains a critical challenge for the commercialization of PGPRs, as microbial inoculants must maintain viability, stability, and efficacy throughout storage and under field conditions. Closely related are **shelf-life issues**, as many beneficial microorganisms lose viability over time, reducing product performance and farmer confidence [154]. Emerging technologies such as microencapsulation and nanoencapsulation enhance the protection of PGPRs against environmental stresses, including UV radiation, desiccation, and high temperatures, thereby improving product shelf life and field performance [154].

The **commercialization of microbial inoculants** is further constrained by limited investment, inadequate large‐scale production technologies, and limited collaboration between researchers and industry, which hinders the translation of promising laboratory findings into commercially viable PGPR products [165]. In addition, **farmer adoption** is also limited by low awareness of PGPR benefits, inconsistent field performance under diverse environmental conditions, and the need for technical guidance on proper application [27]. Moreover, there is a gap between research and practice. Many studies focus on experimental results and overlook the practical needs of farmers, such as cost, ease of use, and reliability. The adoption of PGPR technologies is further constrained by insufficient extension services and ineffective knowledge‐transfer mechanisms [27]. Also, research efforts should be expanded to include understudied plant species in order to broaden the applicability of PGPR technologies. Therefore, sensitization and training programs should be organized for farmers and research institutions to improve awareness and enhance skills related to the application of PGPRs, especially in Africa [27].

Finally, **economic feasibility** influences both manufacturers and growers; the costs associated with product development, registration, quality assurance, and formulation must be balanced against demonstrable economic returns, particularly for medicinal plant production systems where profitability depends on improvements in biomass, secondary metabolite accumulation, and biological activities [27]. Addressing these challenges through harmonized regulations, advanced formulation strategies, robust quality assurance, industry partnerships, farmer education, and comprehensive cost‐benefit evaluations will be essential for the successful commercialization and widespread adoption of PGPRs in medicinal plant production.

## 6. Future Research Directions

The knowledge gaps identified in this review article require a multidisciplinary approach, integrating microbiology, plant physiology, molecular biology, and agronomy. Future research should explore microbiome engineering as a next‐generation approach to PGPR application, integrating multiomics technologies, synthetic microbial consortia, and precision agriculture to develop robust, sustainable, and commercially viable PGPR‐based technologies for enhancing the phytochemical composition and bioactivities of medicinal plants. Future work should include long‐term field studies to assess PGPR’s performance in realistic agricultural conditions. Furthermore, advanced molecular techniques need to be integrated to better understand the mechanisms of PGPR’s effects. This involves the use of omics technologies to study plant–microbe interactions at the genetic and biochemical levels. Future research should focus on the development of multistrain inoculants that can mimic natural microbial communities and yield more stable and consistent results. PGPR technologies require adaptation to local environmental conditions and agricultural practices, which require region‐specific studies. Finally, research and practice should be combined by involving farmers in the research process and improving extension services.

## 7. Conclusions

Several studies have demonstrated that PGPR represents a sustainable and environmentally friendly strategy for improving medicinal plant production. Through direct and indirect mechanisms (Figure 1), PGPRs enhance plant growth and yield, alleviate the effects of biotic and abiotic stresses, and influence the biosynthesis and accumulation of secondary metabolites (Table 2), with corresponding changes in the phytochemical quality and bioactivity of medicinal plants. Despite these promising outcomes, the practical application of PGPRs remains constrained by several knowledge gaps, including an over‐reliance on greenhouse and laboratory studies, limited understanding of the molecular mechanisms underlying PGPR–plant interactions, inconsistent performance across different environmental conditions, insufficient region‐specific research, and challenges associated with the commercialization of microbial inoculants.

Although substantial evidence supports the beneficial effects of PGPRs, relatively few studies have validated these findings under field conditions. Consequently, translating promising experimental results into reliable agricultural practices remains challenging due to environmental variability and inconsistent field performance. Future research should therefore prioritize well‐designed, reproducible field trials using standardized experimental protocols to improve the reliability, reproducibility, and comparability of results. In addition, the development of standardized PGPR formulations with consistent quality, viability, stability, and efficacy will be essential for ensuring reproducible performance, facilitating regulatory approval, and supporting the commercialization and widespread adoption of PGPR technologies.

Future research should also investigate the application of multistrain PGPR consortia, which often exhibit greater potential than individual strains, but whose effectiveness depends on the compatibility between bacterial strains, medicinal plant species, and environmental conditions. Integrating multiomics approaches, including genomics, transcriptomics, proteomics, and metabolomics, together with genetic engineering and microbiome engineering, will provide deeper insights into the molecular mechanisms governing PGPR–plant interactions and facilitate the selection of compatible microbial consortia for specific medicinal plants. Furthermore, because plants in natural environments are typically exposed to multiple simultaneous abiotic stresses rather than individual stress factors, future research should evaluate PGPR performance under combined stress conditions to improve their practical relevance and field applicability. Addressing these knowledge gaps through interdisciplinary research and stronger integration of laboratory, greenhouse, and field studies will accelerate the development of robust, sustainable, and commercially viable PGPR technologies for enhancing the phytochemical composition, bioactivities, and sustainable production of medicinal plants.

## Author Contributions

Conceptualization: Oluwafunbi Christianah Adeleye and Noluyolo Nogemane; writing–original draft: Oluwafunbi Christianah Adeleye; writing–review and editing: Oluwafunbi Christianah Adeleye and Noluyolo Nogemane.

## Funding

This work was supported by UNISA, and we have not received a number for this grant.

## Conflicts of Interest

The authors declare no conflicts of interest.

## Data Availability

Data sharing is not applicable to this article as no datasets were generated or analyzed during the current study.

## References

[1] Rahman S. , Iqbal M. , and Husen A. , Medicinal Plants and Abiotic Stress: An Overview, Medicinal Plants: Their Response to Abiotic Stress. (2023) 1–34, 10.1007/978-981-19-5611-9_1.

[2] Ivanišová E. , Kačániová M. , Savitskaya T. A. , and Grinshpan D. D. , Medicinal Herbs: Important Source of Bioactive Compounds for Food Industry, Herbs and spices-new Processing Technologies, 2021, IntechOpen, 10.5772/intechopen.98819.

[3] World Health Organization , WHO Establishes the Global Centre for Traditional Medicine in India, 2022.

[4] Abubakar M. B. , Usman D. , El-Saber Batiha G. et al., Natural Products Modulating Angiotensin Converting Enzyme 2 (ACE2) as Potential COVID-19 Therapies, Frontiers in Pharmacology. (2021) 12, 10.3389/fphar.2021.629935. PMC8126690 34012391

[5] Bouyahya A. , El Omari N. , Belmehdi O. et al., Pharmacological Investigation of Ajuga Iva Essential Oils Collected at Three Phenological Stages, Flavour and Fragrance Journal. (2021) 36, no. 1, 75–83, 10.1002/ffj.3618.

[6] Jangpangi D. , Patni B. , Chandola V. , and Chandra S. , Medicinal Plants in a Changing Climate: Understanding the Links Between Environmental Stress and Secondary Metabolite Synthesis, Frontiers in Plant Science. (2025) 16, 10.3389/fpls.2025.1587337. PMC12202592 40584865

[7] Jampílek J. and Kráľová K. , Impact of Abiotic Stresses on Production of Secondary Metabolites in Medicinal and Aromatic Plants, New Frontiers in Plant-Environment Interactions: Innovative Technologies and Developments, 2023, Springer Nature Switzerland, 169–252, 10.1007/978-3-031-43729-8_8.

[8] Salam U. , Ullah S. , Tang Z. H. et al., Plant Metabolomics: An Overview of the Role of Primary and Secondary Metabolites Against Different Environmental Stress Factors, Life. (2023) 13, no. 3, 10.3390/life13030706. PMC10051737 36983860

[9] Van Der Walt A. J. and Fitchett J. M. , Extreme Temperature Events (Etes) in South Africa: A Review, South African Geographical Journal. (2021) 104, 1–20, 10.1080/03736245.2021.1907219.

[10] Singh S. , Singh S. , and Awasthi A. , Plant Growth-Promoting Microbial Consortia for the Alleviation of Abiotic Stress in Medicinal and Aromatic Plants, Microbial Based Land Restoration Handbook, 2022, 1, CRC Press, 119–152, 10.1201/9781003147091-7.

[11] Meena M. , Singh S. K. , Swapnil P. , and Kumari P. , The Role of PGPRS in Medicinal Plants Under Abiotic Stress, Medicinal Plants: Their Response to Abiotic Stress, 2023, Springer Nature Singapore, 267–285, 10.1007/978-981-19-5611-9_10.

[12] Kumar R. , Swapnil P. , Meena M. , Selpair S. , and Yadav B. G. , Plant Growth-Promoting Rhizobacteria (PGPR): Approaches to Alleviate Abiotic Stresses for Enhancement of Growth and Development of Medicinal Plants, Sustainability. (2022) 14, no. 23, 10.3390/su142315514.

[13] Iqbal S. , Iqbal M. A. , Li C. , Iqbal A. , and Abbas R. N. , Overviewing Drought and Heat Stress Amelioration—From Plant Responses to Microbe-Mediated Mitigation, Sustainability. (2023) 15, no. 2, 10.3390/su15021671.

[14] Vaghela N. and Gohel S. , Medicinal Plant-Associated Rhizobacteria Enhance the Production of Pharmaceutically Important Bioactive Compounds Under Abiotic Stress Conditions, Journal of Basic Microbiology. (2023) 63, no. 3-4, 308–325, 10.1002/jobm.202200361. 36336634

[15] Van Der Walt A. J. and Fitchett J. M. , Extreme Temperature Events (ETES) in South Africa: A Review, South African Geographical Journal. (2022) 104, no. 1, 70–88, 10.1080/03736245.2021.1907219.

[16] Mbokodo I. , Bopape M. J. , Chikoore H. , Engelbrecht F. , and Nethengwe N. , Heatwaves in the Future Warmer Climate of South Africa, Atmosphere. (2020) 11, no. 7, 10.3390/atmos11070712.

[17] Kiran K. and Sharma G. , Regulation of PGPR-Related Genes in Medicinal Plants in Adverse Conditions, Stress-Responsive Factors and Molecular Farming in Medicinal Plants. (2023) 243–273, 10.1007/978-981-99-4480-4_15.

[18] Mmotla K. , Sibanyoni N. R. , Allie F. et al., Exploring the Intricacies of Plant Growth Promoting Rhizobacteria Interactions: An Omics Review, Annals of Microbiology. (2025) 75, no. 1, 10.1186/s13213-025-01793-y.

[19] Bakka K. and Challabathula D. , Amelioration of Salt Stress Tolerance in Plants by Plant Growth-Promoting Rhizobacteria: Insights From “Omics” Approaches, Plant Microbe Symbiosis, 2020, Springer International Publishing, 303–330, 10.1007/978-3-030-36248-5_16.

[20] Kumar U. , Raj S. , Sreenikethanam A. et al., Multi-Omics Approaches in Plant–Microbe Interactions Hold Enormous Promise for Sustainable Agriculture, Agronomy. (2023) 13, no. 7, 10.3390/agronomy13071804.

[21] Srikanth P. , Sivakumar D. , Sharma A. , and Kaushik N. , Recent Developments in Omics Techniques for Improving Plant Abiotic Stress Using Microbes, International Journal of Environmental Science and Technology. (2025) 22, no. 5, 3787–3810, 10.1007/s13762-024-05957-2.

[22] Salmerón-Manzano E. , Garrido-Cardenas J. A. , and Manzano-Agugliaro F. , Worldwide Research Trends on Medicinal Plants, International Journal of Environmental Research and Public Health. (2020) 17, no. 10, 10.3390/ijerph17103376. PMC7277765 32408690

[23] Mahana A. , Medicinal Properties of the Plant Under Adverse Environmental Conditions, Stress-Responsive Factors and Molecular Farming in Medicinal Plants, 2023, Springer Nature Singapore, 17–28, 10.1007/978-981-99-4480-4_2.

[24] Ahuja A. , Gupta J. , and Gupta R. , Miracles of Herbal Phytomedicines in Treatment of Skin Disorders: Natural Healthcare Perspective, Infectious Disorders-Drug TargetsDisorders. (2021) 21, no. 3, 328–338, 10.2174/1871526520666200622142710. 32568024

[25] Kumari R. , Pandey E. , Bushra S. , Faizan S. , and Pandey S. , Plant Growth Promoting Rhizobacteria (PGPR) Induced Protection: A Plant Immunity Perspective, Physiologia Plantarum. (2024) 176, no. 5, 10.1111/ppl.14495. 39247988

[26] Latif R. and Nawaz T. , Medicinal Plants and Human Health: A Comprehensive Review of Bioactive Compounds, Therapeutic Effects, and Applications, Phytochemistry Reviews. (2025) 25, no. 3, 1–44, 10.1007/s11101-025-10194-7.

[27] Adedeji A. A. , Häggblom M. M. , and Babalola O. O. , Sustainable Agriculture in Africa: Plant Growth-Promoting Rhizobacteria (PGPR) to the Rescue, Scientific African. (2020) 9, 10.1016/j.sciaf.2020.e00492.

[28] Aloo B. N. , Mbega E. R. , Tumuhairwe J. B. , and Makumba B. A. , Advancement and Practical Applications of Rhizobacterial Biofertilizers for Sustainable Crop Production in Sub-Saharan Africa, Agriculture & Food Security. (2021) 10, no. 1, 10.1186/s40066-021-00333-6.

[29] Bennis M. , Kaddouri K. , Badaoui B. et al., Plant Growth Promoting Activities of Pseudomonas sp. and Enterobacter sp. Isolated From the Rhizosphere of Vachellia Gummifera in Morocco, FEMS Microbiology Ecology. (2023) 99, no. 11, 10.1093/femsec/fiad114. 37742210

[30] Koehorst R. R. , The Influence of Phosphorous, Copper, Zinc and Arbuscular Mycorrhiza on Growth, Photosynthetic Processes and Financial Viability of Artemisia Afra Grown in a Simulated Marginial Soil of the Western Cape, 2013, Cape Peninsula University of Technology, https://etd.cput.ac.za/handle/20.500.11838/2025.

[31] Mofokeng M. M. , Araya H. T. , Amoo S. O. et al., Diversity and Conservation Through Cultivation of Hypoxis in Africa—A Case Study of Hypoxis Hemerocallidea, Diversity. (2020) 12, no. 4, 10.3390/d12040122.

[32] Viljoen A. , Chen W. , Mulaudzi N. , and Fouche G. , Aloe Ferox, The South African Herbal Pharmacopoeia, 2023, Academic Press, 57–82, 10.1016/B978-0-323-99794-2.00006-4.

[33] Ndjoubi K. O. , Sharma R. , and Hussein A. A. , Phytochemistry, Ethnopharmacology, and Pharmacology of Lessertia Frutescens (Cancer Bush): A Comprehensive Review, Plants. (2025) 14, no. 14, 10.3390/plants14142086. PMC12299841 40733322

[34] Leonard C. , Chen W. , and Kamatou G. , Warburgia Salutaris, The South African Herbal Pharmacopoeia, 2023, Academic Press, 531–556, 10.1016/B978-0-323-99794-2.00010-6.

[35] Namukobe J. , Kiprotich S. , Adia M. M. , Sekandi P. , and Byamukama R. , African Endangered Plants: Their Phytochemistry, Ethnomedicine, and Conservation Strategies, Bioactive Secondary Metabolites From Medicinal Plants of Africa, 2025, Springer Nature Singapore, 151–180, 10.1007/978-981-96-8590-5_5.

[36] Nogemane N. , Propagation and Quality Assessment for the Introduction of Greyia Radlkoferi Into Commercialization, 2017, University of South Africa, https://www.proquest.com/dissertations-theses/propagation-quality-assessment-introduction/docview/3224600752/se-2?accountid=14648.

[37] Adeleye O. C. and Risenga I. M. , Screening of Phytochemical Profile and Biological Activities in the Leaves, Stems and Roots of South African Portulacaria Afra Using Four Extraction Solvents, Biomedical and Pharmacology Journal. (2022) 15, no. 3, 1561–1572, 10.13005/bpj/2494.

[38] Kisiriko M. , Noleto-Dias C. , Bitchagno G. T. et al., The First Comprehensive Chemical Profiling of Vachellia Gummifera (Willd.) Kyal. & Boatwr., A Plant With Medicinal Value, Chemistry and Biodiversity. (2024) 21, no. 6, 10.1002/cbdv.202400396. 38501581

[39] Nadarajah K. and Abdul Rahman N. S. N. , Plant–Microbe Interaction: Aboveground to Belowground, From the Good to the Bad, International Journal of Molecular Sciences. (2021) 22, no. 19, 10.3390/ijms221910388. PMC8508622 34638728

[40] Das P. P. , Singh K. R. , Nagpure G. et al., Plant-Soil-Microbes: A Tripartite Interaction for Nutrient Acquisition and Better Plant Growth for Sustainable Agricultural Practices, Environmental Research. (2022) 214, 10.1016/j.envres.2022.113821. 35810815

[41] Antoszewski M. , Mierek-Adamska A. , and Dąbrowska G. B. , The Importance of Microorganisms for Sustainable agriculture—A Review, Metabolites. (2022) 12, no. 11, 10.3390/metabo12111100. PMC9694901 36422239

[42] Abdul Malik N. A. , Kumar I. S. , and Nadarajah K. , Elicitor and Receptor Molecules: Orchestrators of Plant Defense and Immunity, International Journal of Molecular Sciences. (2020) 21, no. 3, 10.3390/ijms21030963. PMC7037962 32024003

[43] Dreischhoff S. , Das I. S. , Jakobi M. , Kasper K. , and Polle A. , Local Responses and Systemic Induced Resistance Mediated by Ectomycorrhizal Fungi, Frontiers in Plant Science. (2020) 11, 10.3389/fpls.2020.590063. PMC7767828 33381131

[44] Jain A. , Sarsaiya S. , Singh R. , Gong Q. , Wu Q. , and Shi J. , Omics Approaches in Understanding the Benefits of Plant-Microbe Interactions, Frontiers in Microbiology. (2024) 15, 10.3389/fmicb.2024.1391059. PMC11163067 38860224

[45] Al-Turki A. , Murali M. , Omar A. F. , Rehan M. , and Sayyed R. Z. , Recent Advances in PGPR-Mediated Resilience Toward Interactive Effects of Drought and Salt Stress in Plants, Frontiers in Microbiology. (2023) 14, 10.3389/fmicb.2023.1214845. PMC10565232 37829451

[46] Joshi B. , Jena S. N. , Joshi S. R. , and Bhau B. S. , Recent Advances in Pgprs and Their Application in Imparting Biotic and Abiotic Stress Tolerance in Plants, Industrial Microbiology and Biotechnology: Emerging Concepts in Microbial Technology. (2023) 431–472, 10.1007/978-981-99-2816-3_15.

[47] Khalediyan N. , Weisany W. , and Schenk P. M. , Arbuscular Mycorrhizae and Rhizobacteria Improve Growth, Nutritional Status and Essential Oil Production in Ocimum Basilicum and Satureja Hortensis, Industrial Crops and Products. (2021) 160, 10.1016/j.indcrop.2020.113163.

[48] Khoso M. A. , Wagan S. , Alam I. et al., Impact of Plant Growth-Promoting Rhizobacteria (PGPR) on Plant Nutrition and Root Characteristics: Current Perspective, Plant Stress. (2024) 11, 10.1016/j.stress.2023.100341.

[49] Hasan A. , Tabassum B. , Hashim M. , and Khan N. , Role of Plant Growth Promoting Rhizobacteria (PGPR) as a Plant Growth Enhancer for Sustainable Agriculture: A Review, Bacteria. (2024) 3, no. 2, 59–75, 10.3390/bacteria3020005.

[50] Urooj N. , Bano A. , and Riaz A. , Role of PGPR on the Physiology of Sunflower Irrigated With Produced Water Containing High Total Dissolved Solids (TDS) and its Residual Effects on Soil Fertility, International Journal of Phytoremediation. (2022) 24, no. 6, 567–579, 10.1080/15226514.2021.1957771. 34505549

[51] Wu W. , Chen W. , Liu S. et al., Beneficial Relationships Between Endophytic Bacteria and Medicinal Plants, Frontiers in Plant Science. (2021) 12, 10.3389/fpls.2021.646146. PMC8100581 33968103

[52] Kramer J. , Özkaya Ö. , and Kümmerli R. , Bacterial Siderophores in Community and Host Interactions, Nature Reviews Microbiology. (2020) 18, no. 3, 152–163, 10.1038/s41579-019-0284-4. PMC7116523 31748738

[53] Pršić J. and Ongena M. , Elicitors of Plant Immunity Triggered by Beneficial Bacteria, Frontiers in Plant Science. (2020) 11, 10.3389/fpls.2020.594530. PMC7693457 33304371

[54] S Kumar S. , G Mahale A. , Mifta Faizullah M. , Krishna J. R. , and K Channa T. , A Critical Review on Plant Growth-Promoting Rhizobacteria (PGPR) as a Drought Stress Management Tool, International Journal of Plant & Soil Science. (2021) 33, no. 21, 44–66, https://hal.science/hal-05350228v1, 10.9734/ijpss/2021/v33i2130657.

[55] Roychowdhury R. , Mishra S. , Anand G. et al., Decoding the Molecular Mechanism Underlying Salicylic Acid (SA)‐Mediated Plant Immunity: An Integrated Overview From its Biosynthesis to the Mode of Action, Physiologia Plantarum. (2024) 176, no. 3, 10.1111/ppl.14399. 38894599

[56] Abbasi S. , Sadeghi A. , Omidvari M. , and Tahan V. , The Stimulators and Responsive Genes to Induce Systemic Resistance Against Pathogens: An Exclusive Focus on Tomato as a Model Plant, Biocatalysis and Agricultural Biotechnology. (2021) 33, 10.1016/j.bcab.2021.101993.

[57] Rashid S. , Charles T. C. , and Glick B. R. , Isolation and Characterization of New Plant Growth-Promoting Bacterial Endophytes, Applied Soil Ecology. (2012) 61, 217–224, 10.1016/j.apsoil.2011.09.011.

[58] Khoshru B. , Nosratabad A. F. , Mitra D. et al., Rock Phosphate Solubilizing Potential of Soil Microorganisms: Advances in Sustainable Crop Production, Bacteria. (2023) 2, no. 2, 98–115, 10.3390/bacteria2020008.

[59] Sharma P. , Bakshi P. , Kaur R. et al., Inoculation of Plant-Growth-Promoting Rhizobacteria and Earthworms in the Rhizosphere Reinstates Photosynthetic Attributes and Secondary Metabolites in Brassica Juncea L. Under Chromium Toxicity, Plant and Soil. (2023) 483, no. 1, 573–587, 10.1007/s11104-022-05765-y.

[60] AbdElgawad H. , Magdy Korany S. , Reyad A. M. et al., Synergistic Impacts of Plant-Growth-Promoting Bacteria and Selenium Nanoparticles on Improving the Nutritional Value and Biological Activities of Three Cultivars of Brassica Sprouts, ACS Omega. (2023) 8, no. 29, 26414–26424, 10.1021/acsomega.3c02957. PMC10373182 37521602

[61] Riaz U. , Murtaza G. , Anum W. , Samreen T. , Sarfraz M. , and Nazir M. Z. , Plant Growth-Promoting Rhizobacteria (PGPR) as Biofertilizers and Biopesticides, Microbiota and Biofertilizers: A Sustainable Continuum for Plant and Soil Health, 2020, Springer International Publishing, 181–196, 10.1007/978-3-030-48771-3_11.

[62] Ali S. , Akhtar M. S. , Siraj M. , and Zaman W. , Molecular Communication of Microbial Plant Biostimulants in the Rhizosphere Under Abiotic Stress Conditions, International Journal of Molecular Sciences. (2024) 25, no. 22, 10.3390/ijms252212424. PMC11595105 39596488

[63] Mohammadi M. H. Z. , Panahirad S. , Navai A. , Bahrami M. K. , Kulak M. , and Gohari G. , Cerium Oxide Nanoparticles (CeO2-NPs) Improve Growth Parameters and Antioxidant Defense System in Moldavian Balm (Dracocephalum moldavica L.) Under Salinity Stress, Plant Stress. (2021) 1, 10.1016/j.stress.2021.100006.

[64] Abdelaal K. , AlKahtani M. , Attia K. , Hafez Y. , Király L. , and Künstler A. , The Role of Plant Growth-Promoting Bacteria in Alleviating the Adverse Effects of Drought on Plants, Biology. (2021) 10, no. 6, 10.3390/biology10060520. PMC8230635 34207963

[65] Rao M. J. and Zheng B. , The Role of Polyphenols in Abiotic Stress Tolerance and Their Antioxidant Properties to Scavenge Reactive Oxygen Species and Free Radicals, Antioxidants. (2025) 14, no. 1, 10.3390/antiox14010074. PMC11761259 39857408

[66] Kumar K. , Debnath P. , Singh S. , and Kumar N. , An Overview of Plant Phenolics and Their Involvement in Abiotic Stress Tolerance, Stresses. (2023) 3, no. 3, 570–585, 10.3390/stresses3030040.

[67] Pal P. , Ansari S. A. , Jalil S. U. , and Ansari M. I. , Regulatory Role of Phytohormones in Plant Growth and Development, Plant Hormones in Crop Improvement, 2023, Academic Press, 1–13, 10.1016/B978-0-323-91886-2.00016-1.

[68] Neshat M. , Abbasi A. , Hosseinzadeh A. , Sarikhani M. R. , Dadashi Chavan D. , and Rasoulnia A. , Plant Growth Promoting Bacteria (PGPR) Induce Antioxidant Tolerance Against Salinity Stress Through Biochemical and Physiological Mechanisms, Physiology and Molecular Biology of Plants. (2022) 28, no. 2, 347–361, 10.1007/s12298-022-01128-0. PMC8943118 35400886

[69] Jakubowska Z. , Gradowski M. , and Dobrzyński J. , Role of Plant Growth-Promoting Bacteria (PGPB) in Enhancing Phenolic Compounds Biosynthesis and Its Relevance to Abiotic Stress Tolerance in Plants: A Review, Antonie van Leeuwenhoek. (2025) 118, no. 9, 10.1007/s10482-025-02130-8. PMC12289753 40707699

[70] Zandi P. and Schnug E. , Reactive Oxygen Species, Antioxidant Responses and Implications from a Microbial Modulation Perspective, Biology. (2022) 11, no. 2, 10.3390/biology11020155. PMC8869449 35205022

[71] Gowtham H. G. , Singh S. B. , Shilpa N. et al., Insight Into Recent Progress and Perspectives in Improvement of Antioxidant Machinery Upon PGPR Augmentation in Plants Under Drought Stress: A Review, Antioxidants. (2022) 11, no. 9, 10.3390/antiox11091763. PMC9495777 36139837

[72] Ninkuu V. , Aluko O. O. , Yan J. et al., Phenylpropanoids Metabolism: Recent Insight into Stress Tolerance and Plant Development Cues, Frontiers in Plant Science. (2025) 16, 10.3389/fpls.2025.1571825. PMC12242330 40641862

[73] Tripathi A. , Pandey V. K. , Jain D. et al., An Updated Review on Significance of PGPR-Induced Plant Signalling and Stress Management in Advancing Sustainable Agriculture, Journal of Agriculture and Food Research. (2024) 16, 10.1016/j.jafr.2024.101169.

[74] Kumar A. , Singh S. , Gaurav A. K. , Srivastava S. , and Verma J. P. , Plant Growth-Promoting Bacteria: Biological Tools for the Mitigation of Salinity Stress in Plants, Frontiers in Microbiology. (2020) 11, 10.3389/fmicb.2020.01216. PMC7358356 32733391

[75] Akram W. , Sharif S. , Rehman A. et al., Exploring the Potential of Bacillus Subtilis IS1 and B. Amyloliquificiens IS6 to Manage Salinity Stress and Fusarium Wilt Disease in Tomato Plants by Induced Physiological Responses, Microorganisms. (2024) 12, no. 10, 10.3390/microorganisms12102092. PMC11510684 39458401

[76] Abdelshafy Mohamad O. A. , Ma J. B. , Liu Y. H. et al., Beneficial Endophytic Bacterial Populations Associated With Medicinal Plant Thymus Vulgaris Alleviate Salt Stress and Confer Resistance to Fusarium Oxysporum, Frontiers in Plant Science. (2020) 11, 10.3389/fpls.2020.00047. PMC7033553 32117385

[77] Çakmakçı R. , Haliloglu K. , Türkoğlu A. et al., Effect of Different Plant Growth-Promoting Rhizobacteria on Biological Soil Properties, Growth, Yield and Quality of Oregano (Origanum onites L.), Agronomy. (2023) 13, no. 10, 10.3390/agronomy13102511.

[78] Orozco-Mosqueda M. D. C. , Santoyo G. , and Glick B. R. , Recent Advances in the Bacterial Phytohormone Modulation of Plant Growth, Plants. (2023) 12, no. 3, 10.3390/plants12030606. PMC9921776 36771689

[79] Abdelkhalek A. , Aseel D. G. , Király L. , Künstler A. , Moawad H. , and Al-Askar A. A. , Induction of Systemic Resistance to Tobacco Mosaic Virus in Tomato Through Foliar Application of Bacillus Amyloliquefaciens Strain TBorg1 Culture Filtrate, Viruses. (2022) 14, no. 8, 10.3390/v14081830. PMC9416369 36016452

[80] Stefen D. L. V. , Nunes F. R. , Rodolfo G. R. , Segatto C. , Anastácio T. C. , and Lajus C. R. , How Phytohormones Synthesized by PGPR Affect Plant Growth?, Secondary Metabolites and Volatiles of PGPR in plant-growth Promotion, 2022, Springer International Publishing, 119–131, 10.1007/978-3-031-07559-9_7.

[81] Barooah M. , Goswami G. , and Bora S. S. , Plant Growth-Promoting Rhizobacteria (PGPR): A New Perspective in Abiotic Stress Management of Crop Plants, Microbial Metatranscriptomics Belowground, 2021, Springer Singapore, 655–679, 10.1007/978-981-15-9758-9_31.

[82] Anand G. , Shrivas V. L. , Dubey S. , Bhattacharjee A. , and Sharma S. , Stress-Buster Enterobacter Sp. Alleviates Salinity Stress in Cajanus Cajan Together With Impacting Its Rhizospheric Microbiome, South African Journal of Botany. (2023) 156, 202–212, 10.1016/j.sajb.2023.02.040.

[83] Salwan R. , Sharma M. , Sharma A. , and Sharma V. , Insights Into Plant Beneficial Microorganism-Triggered Induced Systemic Resistance, Plant Stress. (2023) 7, 10.1016/j.stress.2023.100140.

[84] Tiwari R. , Kalra A. , Darokar M. P. et al., Endophytic Bacteria From Ocimum Sanctum and Their Yield Enhancing Capabilities, Current Microbiology. (2010) 60, no. 3, 167–171, 10.1007/s00284-009-9520-x. 19826860

[85] Li Y. , Kong D. , Fu Y. , Sussman M. R. , and Wu H. , The Effect of Developmental and Environmental Factors on Secondary Metabolites in Medicinal Plants, Plant Physiology and Biochemistry. (2020) 148, 80–89, 10.1016/j.plaphy.2020.01.006. 31951944

[86] Compant S. , Cambon M. C. , Vacher C. , Mitter B. , Samad A. , and Sessitsch A. , The Plant Endosphere World–Bacterial Life Within Plants, Environmental Microbiology. (2021) 23, no. 4, 1812–1829, 10.1111/1462-2920.15240. 32955144

[87] Lobato-Ureche M. A. , Pérez-Rodriguez M. M. , Segura D. , Monasterio R. , and Cohen A. C. , Pseudomonas 42P4 and Cellulosimicrobium 60I1 as a Sustainable Approach to Increase Growth, Development, and Productivity in Pepper Plants, Frontiers in Sustainable Food Systems. (2023) 6, 10.3389/fsufs.2022.1111573.

[88] Chanda D. , Sharma G. D. , Ibnat M. , and Jamir T. M. , The Potential Use of Plant Growth Promoting Rhizobacteria (PGPR) for Medicinal Plant Cultivation in Meghalaya: A Review, Current World Environment. (2023) 18, no. 3, 948–955, 10.12944/CWE.18.3.04.

[89] Yu J. B. , Bai M. , Wang C. , Wu H. , and Liang X. , Regulation of Secondary Metabolites Accumulation in Medicinal Plants by Rhizospheric and Endophytic Microorganisms, Medicinal Plant Biology. (2024) 3, no. 1, 10.48130/mpb-0024-0011.

[90] Ali B. , Salicylic Acid: An Efficient Elicitor of Secondary Metabolite Production in Plants, Biocatalysis and Agricultural Biotechnology. (2021) 31, 10.1016/j.bcab.2020.101884.

[91] Roychoudhury A. and Bhowmik R. , Health Benefits of Plant Derived Bioactive Secondary Metabolites as Dietary Constituents, SF Journal of Clinical Case Reports. (2020) 2, no. 1, https://scienceforecastoa.com/.

[92] Akhi M. Z. , Haque M. M. , and Biswas M. S. , Role of Secondary Metabolites to Attenuate Stress Damages in Plants, Antioxidants-Benefits, Sources, Mechanisms of Action, 2021, IntechOpen, 10.5772/intechopen.95495.

[93] Reshi Z. A. , Ahmad W. , Lukatkin A. S. , and Javed S. B. , From Nature to Lab: A Review of Secondary Metabolite Biosynthetic Pathways, Environmental Influences, and in Vitro Approaches, Metabolites. (2023) 13, no. 8, 10.3390/metabo13080895. PMC10456650 37623839

[94] Irfan M. F. , Shafique S. , Shafique S. , Tabassum B. , and Yaseen A. R. , Molecular Pathways in Plant Growth-Promoting Rhizobacteria–Plant Interactions: A Comprehensive Review, Archives of Microbiology. (2026) 208, no. 4, 10.1007/s00203-026-04749-1. 41711964

[95] Kaleh A. M. , Singh P. , Ooi Chua K. , and Harikrishna J. A. , Modulation of Plant Transcription Factors and Priming of Stress Tolerance by Plant Growth-Promoting Bacteria: A Systematic Review, Annals of Botany. (2025) 135, no. 3, 387–402, 10.1093/aob/mcae166. PMC11897433 39279216

[96] Seth K. , Vyas P. , Deora S. , Gupta A. K. , Meena M. , and Swapnil P. , Understanding Plant-Plant Growth-Promoting Rhizobacteria (PGPR) Interactions for Inducing Plant Defense, Plant-Microbe interaction-recent Advances in Molecular and Biochemical Approaches, 2023, Academic Press, 201–226, 10.1016/B978-0-323-91876-3.00010-5.

[97] Lv J. , Yang S. , Zhou W. , Liu Z. , Tan J. , and Wei M. , Microbial Regulation of Plant Secondary Metabolites: Impact, Mechanisms and Prospects, Microbiological Research. (2024) 283, 10.1016/j.micres.2024.127688. 38479233

[98] Asghari B. , Khademian R. , and Sedaghati B. , Plant Growth Promoting Rhizobacteria (PGPR) Confer Drought Resistance and Stimulate Biosynthesis of Secondary Metabolites in Pennyroyal (Mentha pulegium L.) Under Water Shortage Condition, Scientia Horticulturae. (2020) 263, 10.1016/j.scienta.2019.109132.

[99] Del Rosario Cappellari L. , Chiappero J. , Palermo T. B. , Giordano W. , and Banchio E. , Impact of Soil Rhizobacteria Inoculation and Leaf-Chewing Insect Herbivory on Mentha Piperita Leaf Secondary Metabolites, Journal of Chemical Ecology. (2020) 46, no. 7, 619–630, 10.1007/s10886-020-01193-3. 32577987

[100] Liu T. H. , Zhang X. M. , Tian S. Z. , Chen L. G. , and Yuan J. L. , Bioinformatics Analysis of Endophytic Bacteria Related to Berberine in the Chinese Medicinal Plant Coptis Teeta Wall, 3 Biotech. (2020) 10, no. 3, 10.1007/s13205-020-2084-y. PMC7002695 32099737

[101] Makgato M. J. , Araya H. T. , du Plooy C. P. , Mokgehle S. N. , and Mudau F. N. , Effects of Rhizobium Inoculation on N2 Fixation, Phytochemical Profiles and Rhizosphere Soil Microbes of Cancer Bush Lessertia Frutescens (L.), Agronomy. (2020) 10, no. 11, 10.3390/agronomy10111675.

[102] Tripathi A. , Awasthi A. , Singh S. et al., Enhancing Artemisinin Yields Through an Ecologically Functional Community of Endophytes in Artemisia annua, Industrial Crops and Products. (2020) 150, 10.1016/j.indcrop.2020.112375.

[103] Wei M. , Zhang M. , Huang G. , Yuan Y. , Fu C. , and Yu L. , Coculture With Two Bacillus Velezensis Strains Enhances the Growth of Anoectochilus Plants via Promoting Nutrient Assimilation and Regulating Rhizosphere Microbial Community, Industrial Crops and Products. (2020) 154, 10.1016/j.indcrop.2020.112697.

[104] Zhang Y. , Zheng L. , Zheng Y. et al., Insight Into the Assembly of Root-Associated Microbiome in the Medicinal Plant Polygonum Cuspidatum, Industrial Crops and Products. (2020) 145, 10.1016/j.indcrop.2020.112163.

[105] Abdel-Hamid M. S. , Fouda A. , El-Ela H. K. A. , El-Ghamry A. A. , and Hassan S. E. D. , Plant Growth-Promoting Properties of Bacterial Endophytes Isolated From Roots of Thymus Vulgaris L. and Investigate Their Role as Biofertilizers to Enhance the Essential Oil Contents, Biomolecular Concepts. (2021) 12, no. 1, 175–196, 10.1515/bmc-2021-0019. 35041305

[106] Chen J. , Li N. , Chang J. , Ren K. , Zhou J. , and Yang G. E. , Taxonomic Structure of Rhizosphere Bacterial Communities and Its Association With the Accumulation of Alkaloidal Metabolites in Sophora flavescens, Frontiers in Microbiology. (2021) 12, 10.3389/fmicb.2021.781316. PMC8712762 34970241

[107] Costa-Santos M. , Mariz-Ponte N. , Dias M. C. , Moura L. , Marques G. , and Santos C. , Effect of Bacillus spp. and Brevibacillus sp. on the Photosynthesis and Redox Status of Solanum lycopersicum, Horticulturae. (2021) 7, no. 2, 10.3390/horticulturae7020024.

[108] Jiménez-Gómez A. , García-Estévez I. , Escribano-Bailón M. T. , García-Fraile P. , and Rivas R. , Bacterial Fertilizers Based on Rhizobium Laguerreae and Bacillus Halotolerans Enhance Cichorium Endivia L. Phenolic Compound and Mineral Contents and Plant Development, Foods. (2021) 10, no. 2, 10.3390/foods10020424. PMC7919373 33671987

[109] Kang J. P. , Huo Y. , Yang D. U. , and Yang D. C. , Influence of the Plant Growth Promoting Rhizobium Panacihumi on Aluminum Resistance in Panax Ginseng, Journal of Ginseng Research. (2021) 45, no. 3, 442–449, 10.1016/j.jgr.2020.01.001. PMC8134844 34025137

[110] Li Y. , Liu Y. , Zhang H. , Yang Y. , Wei G. , and Li Z. , The Composition of Root-Associated Bacteria and Fungi of Astragalus Mongholicus and Their Relationship With the Bioactive Ingredients, Frontiers in Microbiology. (2021) 12, 10.3389/fmicb.2021.642730. PMC8147693 34046020

[111] Pellegrini M. , Spera D. M. , Ercole C. , and Del Gallo M. , Allium Cepa L. Inoculation With a Consortium of Plant Growth-Promoting Bacteria: Effects on Plants, Soil, and the Autochthonous Microbial Community, Microorganisms. (2021) 9, no. 3, 10.3390/microorganisms9030639. PMC8003585 33808642

[112] Rossi M. , Borromeo I. , Capo C. et al., PGPB Improve Photosynthetic Activity and Tolerance to Oxidative Stress in Brassica napus Grown on Salinized Soils, Applied Sciences. (2021) 11, no. 23, 10.3390/app112311442.

[113] Striganavičiūtė G. , Žiauka J. , Sirgedaitė-Šėžienė V. , and Vaitiekūnaitė D. , Priming of Resistance-Related Phenolics: A Study of Plant-Associated Bacteria and Hymenoscyphus fraxineus, Microorganisms. (2021) 9, no. 12, 10.3390/microorganisms9122504. PMC8707895 34946104

[114] Csorba C. , Rodić N. , Zhao Y. et al., Metabolite Production in Alkanna tinctoria Links Plant Development With the Recruitment of Individual Members of Microbiome Thriving at the Root-Soil Interface, mSystems. (2022) 7, no. 5, e00451–22, 10.1128/msystems.00451-22. PMC9601132 36069453

[115] Eshaghi Gorgi O. , Fallah H. , Niknejad Y. , and Barari Tari D. , Effect of Plant Growth Promoting Rhizobacteria (PGPR) and Mycorrhizal Fungi Inoculations on Essential Oil in Melissa officinalis L. Under Drought Stress, Biologia. (2022) 77, no. 1, 11–20, 10.1007/s11756-021-00919-2.

[116] He D. , Singh S. K. , Peng L. et al., Flavonoid-Attracted Aeromonas sp. From the Arabidopsis Root Microbiome Enhances Plant Dehydration Resistance, The ISME Journal. (2022) 16, no. 11, 2622–2632, 10.1038/s41396-022-01288-7. PMC9561528 35842464

[117] Lin Y. , Zhang H. , Li P. , Jin J. , and Li Z. , The Bacterial Consortia Promote Plant Growth and Secondary Metabolite Accumulation in Astragalus mongholicus Under Drought Stress, BMC Plant Biology. (2022) 22, no. 1, 10.1186/s12870-022-03859-4. PMC9541091 36203134

[118] Liu S. Y. , Wang Q. Q. , Lei Y. H. et al., Elucidating the Interaction of Rhizosphere Bacteria and Environmental Factors in Influencing Active Ingredient Content of Lycium Barbarum Fruit in China, Journal of Applied Microbiology. (2022) 132, no. 5, 3783–3796, 10.1111/jam.15502. 35191572

[119] Polito G. , Semenzato G. , Del Duca S. et al., Endophytic Bacteria and Essential Oil From Origanum vulgare ssp. Vulgare Share Some Vocs With an Antibacterial Activity, Microorganisms. (2022) 10, no. 7, 10.3390/microorganisms10071424. PMC9320186 35889143

[120] Singh B. , Sahu P. M. , Aloria M. , Reddy S. S. , Prasad J. , and Sharma R. A. , Azotobacter Chroococcum and Pseudomonas Putida Enhance Pyrroloquinazoline Alkaloids Accumulation in Adhatoda Vasica Hairy Roots by Biotization, Journal of Biotechnology. (2022) 353, 51–60, 10.1016/j.jbiotec.2022.05.011. 35691257

[121] Jagtap R. R. , Mali G. V. , Waghmare S. R. , Nadaf N. H. , Nimbalkar M. S. , and Sonawane K. D. , Impact of Plant Growth Promoting Rhizobacteria Serratia Nematodiphila RGK and Pseudomonas plecoglossicida RGK on Secondary Metabolites of Turmeric Rhizome, Biocatalysis and Agricultural Biotechnology. (2023) 47, 10.1016/j.bcab.2023.102622.

[122] Kang S. M. , Hoque M. I. U. , Woo J. I. , and Lee I. J. , Mitigation of Salinity Stress on Soybean Seedlings Using Indole Acetic Acid-Producing Acinetobacter Pittii YNA40, Agriculture. (2023) 13, no. 5, 10.3390/agriculture13051021.

[123] Liu Z. , Ma Y. , Lv X. et al., Abiotic Factors and Endophytes Co-Regulate Flavone and Terpenoid Glycoside Metabolism in Glycyrrhiza Uralensis, Applied Microbiology and Biotechnology. (2023) 107, no. 7, 2671–2688, 10.1007/s00253-023-12441-3. PMC10033487 36864204

[124] Lyu D. , Backer R. , Berrué F. , Martinez-Farina C. , Hui J. P. , and Smith D. L. , Plant Growth-Promoting Rhizobacteria (PGPR) With Microbial Growth Broth Improve Biomass and Secondary Metabolite Accumulation of Cannabis Sativa L, Journal of Agricultural and Food Chemistry. (2023) 71, no. 19, 7268–7277, 10.1021/acs.jafc.2c06961. PMC10197126 37130078

[125] Pérez-García L. A. , Sáenz-Mata J. , Fortis-Hernández M. , Navarro-Muñoz C. E. , Palacio-Rodríguez R. , and Preciado-Rangel P. , Plant-Growth-Promoting Rhizobacteria Improve Germination and Bioactive Compounds in Cucumber Seedlings, Agronomy. (2023) 13, no. 2, 10.3390/agronomy13020315.

[126] Semenzato G. , Del Duca S. , Vassallo A. et al., Genomic, Molecular, and Phenotypic Characterization of Arthrobacter sp. OVS8, an Endophytic Bacterium Isolated From and Contributing to the Bioactive Compound Content of the Essential Oil of the Medicinal Plant Origanum vulgare L, International Journal of Molecular Sciences. (2023) 24, no. 5, 10.3390/ijms24054845. PMC10002853 36902273

[127] Tariq S. , Bano A. , and Khan N. , Comparative Analysis of Various Strains of Plant Growth Promoting Rhizobacteria on the Physiology of Garlic (Allium sativum), Natural Science. (2023) 15, no. 03, 79–90, 10.4236/ns.2023.153007.

[128] Ferreira M. J. , Veríssimo A. C. , Pinto D. C. et al., Engineering the Rhizosphere Microbiome With Plant Growth Promoting Bacteria for Modulation of the Plant Metabolome, Plants. (2024) 13, no. 16, 10.3390/plants13162309. PMC11360282 39204745

[129] Kisiel A. , Miller T. , Łobodzińska A. , and Rybak K. , Biosynthesis of Phenolic Compounds of Medicago truncatula After Inoculation With Selected PGPR Strains, International Journal of Molecular Sciences. (2024) 25, no. 23, 10.3390/ijms252312684. PMC11641612 39684404

[130] Nafees M. , Ullah S. , and Ahmed I. , Plant Growth-Promoting Rhizobacteria and Biochar as Bioeffectors and Bioalleviators of Drought Stress in Faba Bean (Vicia faba L.), Folia Microbiologica. (2024) 69, no. 3, 653–666, 10.1007/s12223-023-01103-6. 37940775

[131] Ng C. W. W. , Yan W. H. , Xia Y. T. , Tsim K. W. K. , and To J. C. T. , Plant Growth-Promoting Rhizobacteria Enhance Active Ingredient Accumulation in Medicinal Plants at Elevated CO2 and are Associated With Indigenous Microbiome, Frontiers in Microbiology. (2024) 15, 10.3389/fmicb.2024.1426893. PMC11381388 39252828

[132] Sridhar D. , Alherwairini S. S. , Eswaran S. U. D. , Barasarathi J. , Lalitha S. , and Sayyed R. , The Soil Microbiome Enhances Sesame Growth and Oil Composition, and Soil Nutrients Under Saline Conditions, Scientific Reports. (2025) 15, no. 1, 10.1038/s41598-025-15589-2. PMC12339991 40790085

[133] Kousar B. , Bano A. , and Khan N. , PGPR Modulation of Secondary Metabolites in Tomato Infested With Spodoptera litura, Agronomy. (2020) 10, no. 6, 10.3390/agronomy10060778.

[134] Yue L. , Uwaremwe C. , Tian Y. et al., Bacillus Amyloliquefaciens Rescues Glycyrrhizic Acid Loss Under Drought Stress in Glycyrrhiza uralensis by Activating the Jasmonic Acid Pathway, Frontiers in Microbiology. (2022) 12, 10.3389/fmicb.2021.798525. PMC8966401 35368293

[135] El-Ballat E. M. , Elsilk S. E. , Ali H. M. , Ali H. E. , Hano C. , and El-Esawi M. A. , Metal-Resistant PGPR Strain Azospirillum brasilense EMCC1454 Enhances Growth and Chromium Stress Tolerance of Chickpea (Cicer arietinum L.) by Modulating Redox Potential, Osmolytes, Antioxidants, and Stress-Related Gene Expression, Plants. (2023) 12, no. 11, 10.3390/plants12112110. PMC10255068 37299089

[136] Singh S. , Chanotiya C. S. , Singh A. , Vajpayee P. , and Kalra A. , Role of ACC-Deaminase Synthesizing Trichoderma Harzianum and Plant Growth-Promoting Bacteria in Reducing Salt-Stress in Ocimum Sanctum, Physiology and Molecular Biology of Plants. (2023) 29, no. 6, 815–828, 10.1007/s12298-023-01328-2. PMC10382467 37520812

[137] Su J. , Wang Y. , Bai M. et al., Soil Conditions and the Plant Microbiome Boost the Accumulation of Monoterpenes in the Fruit of Citrus Reticulata ‘Chachi, Microbiome. (2023) 11, no. 1, 10.1186/s40168-023-01504-2. PMC10044787 36973820

[138] Kennedy D. O. and Wightman E. L. , Herbal Extracts and Phytochemicals: Plant Secondary Metabolites and the Enhancement of Human Brain Function, Advances in Nutrition. (2011) 2, no. 1, 32–50, 10.3945/an.110.000117. PMC3042794 22211188

[139] Kandar C. C. , Secondary Metabolites From Plant Sources, Bioactive Natural Products for Pharmaceutical Applications, 2020, Springer International Publishing, 329–377, 10.1007/978-3-030-54027-2_10.

[140] Lv Z. Y. , Sun W. J. , Jiang R. et al., Phytohormones Jasmonic Acid, Salicylic Acid, Gibberellins, and Abscisic Acid are Key Mediators of Plant Secondary Metabolites, World Journal of Traditional Chinese Medicine. (2021) 7, no. 3, 307–325, 10.4103/wjtcm.wjtcm_20_21.

[141] Aggag S. , Gaber M. , Elmesseiry S. , and El-Tanbouly R. , Synergistic Effects of Bacillus Velezensis and Indole Acetic Acid on Alkaloids Pathway in Catharanthus roseus, Egyptian Academic Journal of Biological Sciences, H. Botany. (2026) 17, no. 1, 25–42, 10.21608/eajbsh.2026.484982.

[142] Nandy S. , Das T. , and Dey A. , Role of Jasmonic Acid and Salicylic Acid Signaling in Secondary Metabolite Production, Jasmonates and Salicylates Signaling in Plants. (2021) 87–113, 10.1007/978-3-030-75805-9_5.

[143] Wen L. , Zhao Y. , Jiang Y. et al., Identification of a Flavonoid C-Glycoside as Potent Antioxidant, Free Radical Biology and Medicine. (2017) 110, 92–101, 10.1016/j.freeradbiomed.2017.05.027. 28587909

[144] Teoh E. S. , Medicinal Orchids of Asia, 2016, 16, Springer, 10.1007/978-3-319-24274-3.

[145] Sun X. , Zhang X. , Zhang G. et al., Environmental Response to Root Secondary Metabolite Accumulation in Paeonia Lactiflora: Insights From Rhizosphere Metabolism and Root-Associated Microbial Communities, Microbiology Spectrum. (2022) 10, no. 6, e02800–e02822, 10.1128/spectrum.02800-22. PMC9769548 36318022

[146] Peñuelas J. , Asensio D. , Tholl D. et al., Biogenic Volatile Emissions From the Soil, Plant, Cell and Environment. (2014) 37, no. 8, 1866–1891, 10.1111/pce.12340. 24689847

[147] Chouhan R. , Ahmed S. , and Gandhi S. G. , Plant Volatile Organic Compounds and Neuroregenerative Health, Botanical Leads for Drug Discovery, 2020, Springer Singapore, 105–136, 10.1007/978-981-15-5917-4_6.

[148] Laller R. , Khosla P. K. , Negi N. et al., Bacterial Volatiles as Pgprs: Inducing Plant Defense Mechanisms During Stress Periods, South African Journal of Botany. (2023) 159, 131–139, 10.1016/j.sajb.2023.05.041.

[149] Thakur R. , Dhar H. , Mathew S. , and Gulati A. , PGPR Inoculants Journey From Lab to Land: Challenges and Limitations, Microbiological Research. (2024) 289, no. 127910, 10–1016, 10.1016/j.micres.2024.127910. 39303413

[150] Zandalinas S. I. , Peláez-Vico M. Á. , Sinha R. , Pascual L. S. , and Mittler R. , The Impact of Multifactorial Stress Combination on Plants, Crops, and Ecosystems: How Should we Prepare for What Comes Next?, The Plant Journal. (2024) 117, no. 6, 1800–1814, 10.1111/tpj.16557. 37996968

[151] Langstroff A. , Heuermann M. C. , Stahl A. , and Junker A. , Opportunities and Limits of Controlled-Environment Plant Phenotyping for Climate Response Traits, Theoretical and Applied Genetics. (2022) 135, no. 1, 1–16, 10.1007/s00122-021-03892-1. PMC8741719 34302493

[152] Sanam T. , S T. , Nerella S. G. , Ningoji S. N. , and Desai S. , Correlation and Regression Models of Tomato Yield in Response to Plant Growth by Different Bacterial Inoculants and Inoculation Methods, Agronomy Journal. (2022) 114, no. 1, 452–460, 10.1002/agj2.20951.

[153] Naqvi S. A. H. , Umar U. U. D. , and Rehman A. U. , Host-Specific Multi-Strain PGPR Consortium Delivers Superior Bacterial Leaf Blight Control and Yield Enhancement in Rice Through Integrated Seed Priming and Foliar Applications, European Journal of Plant Pathology. (2026) 174, no. 8, 1–27, 10.1007/s10658-026-03212-7.

[154] Yang P. , Condrich A. , Scranton S. , Hebner C. , Lu L. , and Ali M. A. , Utilizing Plant Growth-Promoting Rhizobacteria (PGPR) to Advance Sustainable Agriculture, Bacteria. (2024) 3, no. 4, 434–451, 10.3390/bacteria3040030.

[155] de Andrade L. A. , Santos C. H. B. , Frezarin E. T. , Sales L. R. , and Rigobelo E. C. , Plant Growth-Promoting Rhizobacteria for Sustainable Agricultural Production, Microorganisms. (2023) 11, no. 4, 10.3390/microorganisms11041088. PMC10146397 37110511

[156] Maheshwari D. K. , Das A. , Dheeman S. , and Pandey P. , An Overall Insight Into the Attributes, Interactions, and Future Applications of “Microbial Consortium” for Plant Growth Promotion With Contemporary Approaches, Sustainable Agrobiology: Design and Development of Microbial Consortia. (2023) 3–22, 10.1007/978-981-19-9570-5_1.

[157] Márquez D. G. P. , Munaro D. , and Uarrota V. G. , Metabolomics as a Tool to Study Volatile Organic Compounds Produced by Plant Growth-Promoting Rhizobacteria, Secondary Metabolites and Volatiles of PGPR in Plant-Growth Promotion, 2022, Springer International Publishing, 21–44, 10.1007/978-3-031-07559-9_2.

[158] Sahoo A. , Yadav G. , Mehta T. , Meena M. , and Swapnil P. , Omics-Driven Insights Into Plant growth-promoting Microorganisms for Sustainable Agriculture, Discover Sustainability. (2025) 6, no. 1, 10.1007/s43621-025-01582-2.

[159] Mesele E. , Gebreslassie G. , Yaekob A. T. , and Semere T. , Molecular Mechanisms of Plant Growth–Promoting Rhizobacteria (PGPR) in Biofertilization and Disease Suppression of Crops, International Journal of Agronomy. (2026) 2026, no. 1, 10.1155/ioa/9398708.

[160] Shu W. S. and Huang L. N. , Microbial Diversity in Extreme Environments, Nature Reviews Microbiology. (2022) 20, no. 4, 219–235, 10.1038/s41579-021-00648-y. 34754082

[161] Bhardwaj I. , Kumar V. , Singh S. et al., Evaluation of Stress-Tolerant Serratia and Enterobacter as PGPR for Nutrient Solubilization and Dose-Dependent Bioformulation to Enhance Tomato Seedlings, Plants. (2025) 14, no. 14, 10.3390/plants14142154. PMC12299828 40733392

[162] Hoveka L. N. , van Der Bank M. , Bezeng B. S. , and Davies T. J. , Identifying Biodiversity Knowledge Gaps for Conserving South Africa’s Endemic Flora, Biodiversity & Conservation. (2020) 29, no. 9, 2803–2819, 10.1007/s10531-020-01998-4.

[163] Danesh Y. R. and Pellegrini M. , The Role of Pgprs in Enhancing the Quality and Production of Medicinally Important Plants, Phytoceuticals for Sustainable Medication. (2026) 151–172, 10.1007/978-981-95-7588-6_6.

[164] Basu A. , Prasad P. , Das S. N. et al., Plant Growth Promoting Rhizobacteria (PGPR) as Green Bioinoculants: Recent Developments, Constraints, and Prospects, Sustainability. (2021) 13, no. 3, 10.3390/su13031140.

[165] Shah A. , Nazari M. , Antar M. et al., PGPR in Agriculture: A Sustainable Approach to Increasing Climate Change Resilience, Frontiers in Sustainable Food Systems. (2021) 5, 10.3389/fsufs.2021.667546.

